# Pilot study of probiotic/colostrum supplementation on gut function in children with autism and gastrointestinal symptoms

**DOI:** 10.1371/journal.pone.0210064

**Published:** 2019-01-09

**Authors:** Megan R. Sanctuary, Jennifer N. Kain, Shin Yu Chen, Karen Kalanetra, Danielle G. Lemay, Destanie R. Rose, Houa T. Yang, Daniel J. Tancredi, J. Bruce German, Carolyn M. Slupsky, Paul Ashwood, David A. Mills, Jennifer T. Smilowitz, Kathleen Angkustsiri

**Affiliations:** 1 Department of Nutrition, University of California, Davis, California, United States of America; 2 Department of Neurobiology, Physiology and Behavior, University of California, Davis, California, United States of America; 3 Department of Food Science and Technology, University of California, Davis, California, United States of America; 4 USDA ARS Western Human Nutrition Research Center, Davis, California, United States of America; 5 Genome Center, University of California, Davis, California, United States of America; 6 MIND Institute, University of California Davis, Sacramento, California, United States of America; 7 Department of Pediatrics, University of California School of Medicine, Sacramento, California, United States of America; 8 Foods for Health Institute, University of California, Davis, California, United States of America; TNO, NETHERLANDS

## Abstract

Over half of all children with autism spectrum disorders (ASD) have gastrointestinal (GI) co-morbidities including chronic constipation, diarrhea, and irritable bowel syndrome. The severity of these symptoms has been correlated with the degree of GI microbial dysbiosis. The study objective was to assess tolerability of a probiotic (*Bifidobacterium infantis*) in combination with a bovine colostrum product (BCP) as a source of prebiotic oligosaccharides and to evaluate GI, microbiome and immune factors in children with ASD and GI co-morbidities. This pilot study is a randomized, double blind, controlled trial of combination treatment (BCP + *B*. *infantis*) vs. BCP alone in a cross-over study in children ages 2–11 with ASD and GI co-morbidities (n = 8). This 12-week study included 5 weeks of probiotic-prebiotic supplementation, followed by a two-week washout period, and 5 weeks of prebiotic only supplementation. The primary outcome of tolerability was assessed using validated questionnaires of GI function and atypical behaviors, along with side effects. Results suggest that the combination treatment is well-tolerated in this cohort. The most common side effect was mild gassiness. Some participants on both treatments saw a reduction in the frequency of certain GI symptoms, as well as reduced occurrence of particular aberrant behaviors. Improvement may be explained by a reduction in IL-13 and TNF-α production in some participants. Although limited conclusions can be drawn from this small pilot study, the results support the need for further research into the efficacy of these treatments.

## Introduction

Autism spectrum disorder (ASD) refers to a group of heterogeneous neurodevelopmental disorders characterized by social deficits, repetitive and stereotypical behaviors, insistence on routines and communication impairments [1–3]. It is now evident that gastrointestinal (GI) abnormalities are characteristic of a substantial number of children with ASD. An estimated 50% or more of these children are affected with the prevalence of symptoms four times greater than in children without ASD [4]. The most commonly reported symptoms include chronic constipation, diarrhea, and abdominal pain [4–6]. In addition, GI microbial dysbiosis, an imbalance in the organisms that make up the gut microbiota, has been documented in multiple studies of children with ASD [5, 7–14]. While a consensus on the differences in the ASD microbiota compared to typically developing children has yet to be established, there are several examples of how they differ depending on the populations observed. Most studies demonstrate that children with ASD have more bacterial diversity [10, 14] and possess lower overall abundance of potentially beneficial taxa, such as *Bifidobacterium* and *Akkermansia*, [5, 10, 11], although some populations appear to differ from this trend [10, 13]. Many reports show that children with ASD have higher counts of potentially pathogenic *Clostridia* than typically developing children [7–10, 12], although this is not universally true [13]. Further, an abnormal ratio of Firmicutes to Bacteroidetes [12] has been observed as well as increased levels of certain detrimental taxa, such as Proteobacteria [10, 12]. ASD participants with GI symptoms display higher measures of irritability, anxiety and social withdrawal [15]. The degree of this dysbiosis has been correlated with both GI symptoms [9] and ASD symptom severity [11].

Furthermore, children with ASD and gastrointestinal symptoms also present with immune imbalances in the gut that could be associated with abnormal host responses to microbial dysbiosis and impaired gut barrier integrity [16–19]. These immune imbalances represent a unique gastrointestinal immunopathology that is characterized by ileal nodular lymphoid hyperplasia and immune cell infiltration throughout the GI tract [18]. In addition, colonic lesions containing high numbers of infiltrating γδ and CD8^+^ T cells with associated epithelial damage is reported in children with autism [20]. Increased pro-inflammatory (TNF-α and IL-6) but decreased regulatory (IL-10) cytokine production in mucosal lamina propria and epithelial lymphocytes was observed in ASD children with GI symptoms compared to typically developing controls matched on GI symptoms [21, 22]. These cytokine profiles may reflect abnormal responses to bacteria or food antigens. High prevalence of atopic disease, including food allergy, is seen in children with ASD [23]. Therefore, interventions aimed at resolving intestinal dysbiosis could not only help to reduce the frequency and severity of GI symptoms in children with ASD but may also help balance the immune system and potentially improve certain behavioral symptoms as well [24].

Children with ASD have been reported to generally consume <50% of the daily recommended intake of fiber [25]. In addition, suboptimal breastfeeding practices, including non-intake of colostrum and short duration of breastfeeding, have also been shown to be associated with ASD [26]. Interestingly, oligosaccharides, the third most abundant component of human milk, serve to selectively promote the growth of bifidobacteria in the gut while providing limited nutritional support to the infant directly [27–29]. The probiotic *Bifidobacterium longum* subsp. *infantis* (*B*. *infantis*) is considered to be beneficial to GI health. It has been found to dominate the guts of healthy breastfed infants [30] and thus is associated with numerous beneficial health outcomes [31, 32]. It has also been shown *in vitro* to improve gut barrier integrity [33] and reduce expression of inflammatory genes in intestinal epithelial cells [34]. This bacteria grows exceptionally well in the presence of complex milk oligosaccharides to the exclusion of other potentially harmful bacteria [27–29]. Bovine colostrum not only contains a limited amount of milk oligosaccharides that may serve as a selective food source (prebiotic) and promote the growth of this particular bacteria [35, 36], but also contains an abundance of immune proteins, such as immunoglobulins, lactoferrin and numerous cytokines. These proteins have been shown to resist digestion [37, 38] in order to be biologically active in the gut [39] and can further modulate the microbiota [40] and the immune system [40–42]. In addition, many of these immune proteins are heavily glycosylated [43, 44], which may also provide a prebiotic effect as these sugars can be cleaved by bacterial glycosidases [45, 46]. Therefore, concurrent supplementation with both the probiotic *B*. *infantis* and bovine colostrum product (BCP) as a source of immune factors and prebiotic glycans could alter the microbiota to a more beneficial composition in order to improve gut health in children with ASD and GI symptoms.

Combination probiotic-BCP supplementation has yet to be studied in children with ASD and GI symptoms in a systematic and controlled trial. Many children on the autism spectrum have sensory sensitivities resulting in restricted and selective diets [47] which can often make oral administration of medications or supplements challenging. Therefore, this study first aims to establish supplement tolerability to establish trial feasibility to design a larger future study of a combined probiotic–BCP supplement. Primary outcome measures include changes to the intestinal microbiota as well as supplement tolerability. GI symptoms and any behavioral changes were monitored throughout the study as measures of tolerability. Secondary outcome measures included changes to the intestinal microbiota, changes in peripheral blood mononuclear cell (PBMC) cytokine expression as well as changes in host and microbial metabolism through metabolomic analysis of plasma, urine and stool.

## Materials and methods

### Study participants and protocol

The study was a double-blind, crossover, randomized clinical trial (RCT) conducted at the Medical Investigation of Neurodevelopmental Disorders (MIND) Institute and University of California Davis Medical Center, Sacramento, California, from 6/6/14 to 11/16/15.

Children with a previous diagnosis of ASD, ages 2–11 with a history of frequent gastrointestinal symptoms including chronic constipation, diarrhea, and/or irritable bowel syndrome (IBS) were recruited for this study. Participants were recruited from local community events, as well as from previous studies conducted at the MIND Institute. This study protocol received ethical approval from the Institutional Review Board of the University of California at Davis (Approval#: 450072) on 6/16/2013. The authors confirm that all ongoing and related trials for this drug/intervention are registered and this trial was registered on ClinicalTrials.gov Identifier: NCT02086110. Participants were provided with a complete description of the study and written informed consent was obtained from all parents/guardians prior to study enrollment.

**Fig 1:**
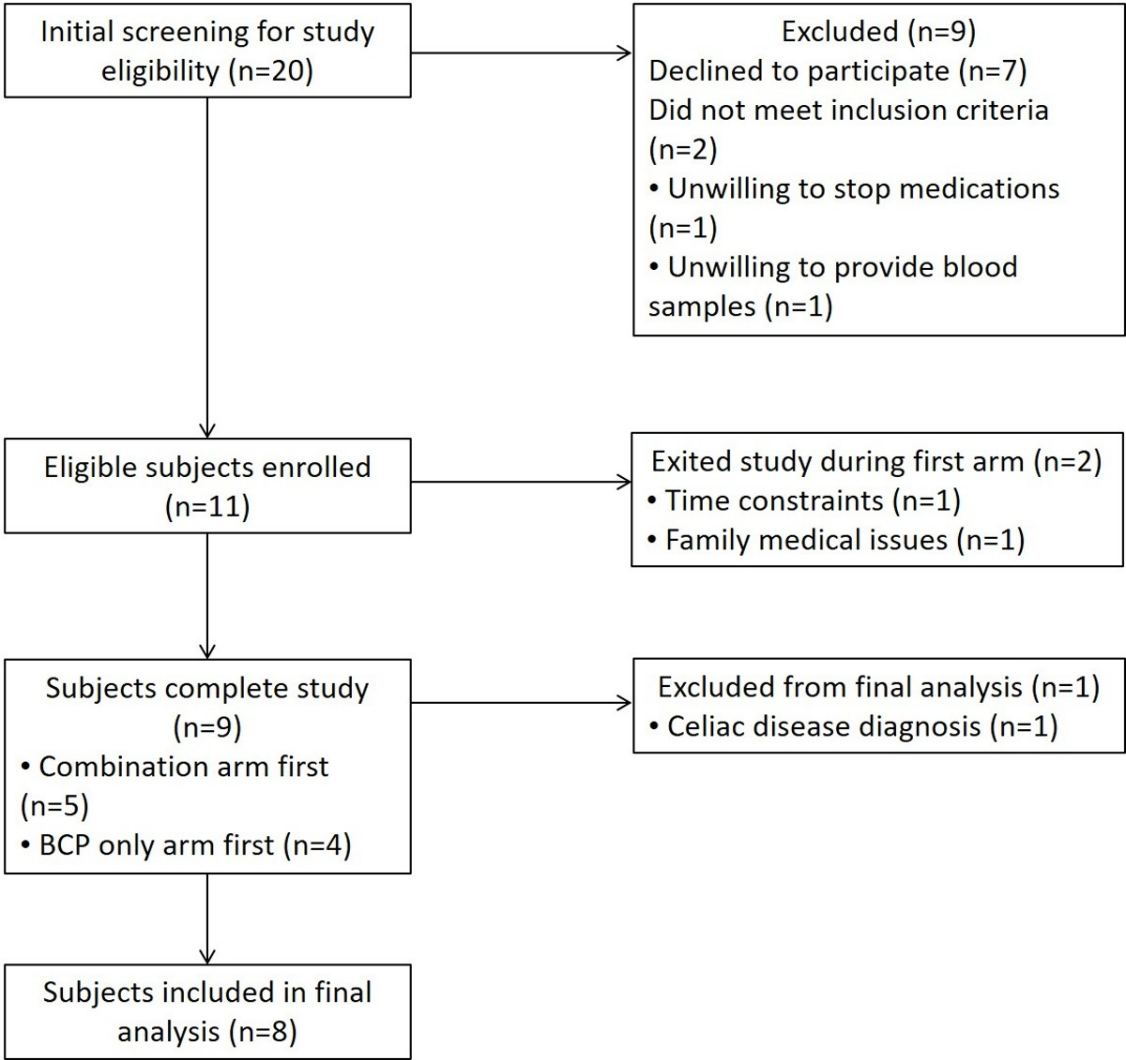
Study subject enrollment flow chart. Diagram describing the number of participants screened, randomized into treatment groups, screen-failed, withdrew throughout the study period and included in the final analysis.

The inclusion criteria included a previous diagnosis of ASD based on documented ADOS (Autism Diagnostic Observation Schedule [48]) scores as well as meeting criteria for functional constipation, functional diarrhea, and/or irritable bowel syndrome (IBS) based on the Questionnaire on Pediatric Gastrointestinal Symptoms-Rome III Version (QPGS-RIII) diagnostic criteria [49] as completed by the parent. All participants were screened via parental interview for current and past physical illnesses and medication use over the past 3 months. Participants were excluded from the study if they had a self-reported history of milk protein allergy. Exclusionary medical conditions included compromised immunity, GI disease (*e*.*g*. inflammatory bowel disease, celiac disease, short gut), systemic steroid, antifungal or antibiotic use within the past 3 months, BMI<5^th^ percentile for age, and individuals on medically prescribed diets or supplements (including probiotic use within the past month) other than a standard multivitamin. Children with other co-morbid disorders, such as uncontrolled seizure disorder, genetic disorders (*e*.*g*. fragile X syndrome, tuberous sclerosis) or other serious medical condition as determined by the study physician were also excluded (Fig 1). Participant demographics are provided in Table 1.

**Table 1:** Participant demographics at baseline.

Characteristic	Value
Average age at enrollment (years ± SD)	6.8 ± 2.4
Age range (years)	3.9–10.9
Male:female, % (n)	87.5:12.5 (7:1)
Average Initial ABAS-II Composite Score (mean ± SD)	57 ± 11
Race (White/African-American), % (n)	87.5 (7)/12.5 (1)
Ethnicity (Non-Hispanic/Hispanic or Latino), % (n)	75 (6)/25 (2)
Initial Constipation/Diarrhea/Both based on QPGS-RIII, % (n)	25 (2)/62.5 (5)/12.5 (1)
Initial Irritable Bowel Syndrome, % (n)	62.5 (5)
Initial Vomiting, % (n)	25 (2)
Initial Gas/bloating, % (n)	87.5 (7)
Picky Eater, % (n)	87.5 (7)
Low Baseline BMI (5-10^th^ percentile), % (n)	25 (2)

**Fig 2:**
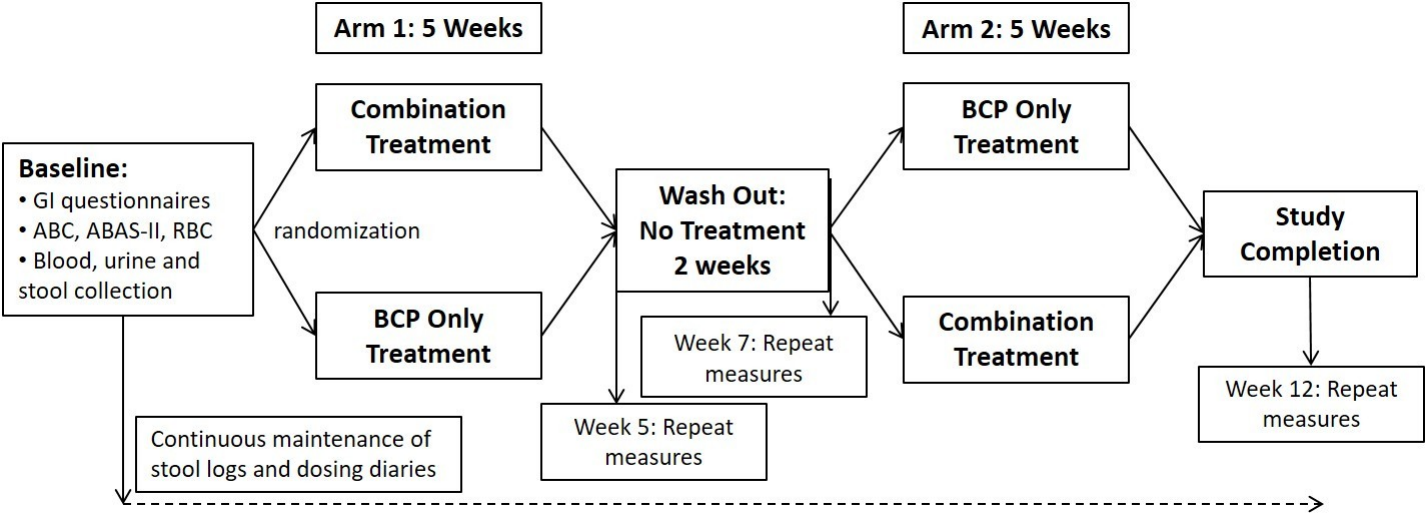
Study design flow chart. Diagram describing the study design including outcomes measured, timing and duration of the study period.

**Fig 3:**
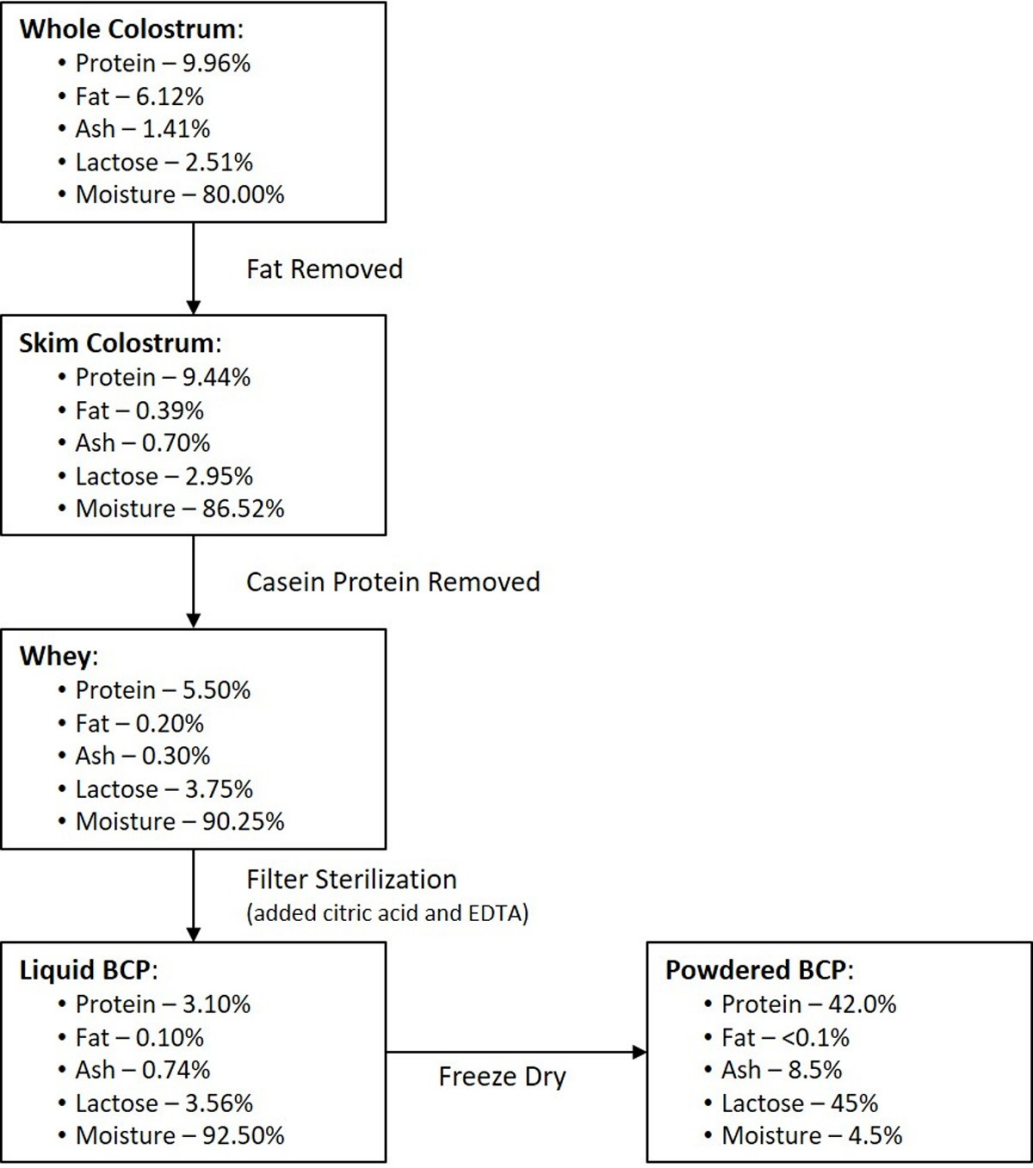
BCP work flow chart. Diagram describing work flow for bovine colostrum product (Imucon) production. The dried Imucon product (powdered BCP) was produced from the commercially available liquid Imucon product (liquid BCP) for specific use in the current study.

All participants (n = 11) were randomly assigned to receive either the BCP only or the combination (BCP + *Bifidobacterium infantis*) during the first or second arm of the study. After the first 5-week arm, participants underwent a 2-week wash out period in which no treatment was received, followed by the second 5-week arm. Each participant received both treatments, but were randomized as to the order of treatment. Subjects were enrolled by the study coordinator, MRS, after eligibility was verified by the study physician, KA. Randomization was performed by the Investigational Drug Service (IDS) at the UC Davis Medical Center so that all study personnel and participants were blinded as to the order of treatment. The crossover study design allowed each child to serve as their own control for purposes of quantifying the effects of combination treatment on fecal microbiota changes. An analysis of ten participants allows for 80% power to detect differences of magnitude 1.94 RMSE, under two-sided testing (α = 0.05) of the null hypothesis that the effect size is 0. Previous studies have measured similar bifidogenic responses in infants, for whom the within-infant correlations have ranged as high as 40%. Participants were asked to visit the MIND Institute four times, once at the beginning and end of each study arm (Fig 2). Both treatments were provided in powder form with identical taste and texture to preserve the blinding of the study. The colostrum powder dose administered in this study was 0.15 g/lb body weight per day. Thus, the range of administered BCP was 5.1–10.8 g per day depending on the child’s weight. This dose provides approximately 12.5% of the RDA for dietary protein and 0.319–0.675 g fiber per day and was selected to slightly increase fiber intake while limiting increase in protein intake. The RDA for fiber intake in children varies by age group in children so weight was used as a surrogate for differences in dietary intake amongst this cohort [50]. In addition, similar bovine colostrum products (defatted, reduced casein, whey proteins, lactose, growth factors) have been previously tested outside the US. One study using bovine colostrum based products tested the effect on HIV induced diarrhea. Participants were treated for 4 weeks and given 50 grams of bovine colostrum twice a day [51]. In the current study, the bovine colostrum product (Imucon) was produced by defatting, decaseinating and sterile-filtering raw bovine colostrum (Fig 3) and was kindly provided by Sterling Technologies (Table 2) and is commercially available in its liquid form (Colostrum Gold^TM^). A certificate of analysis is available for the final product confirming the safety of the product. The product was tested and found to be negative for Escherichia coli, Salmonella, Listeria, coagulase positive Staphylococcus and antibiotic residue. The product contained negligible amounts of other microbes (<10 cfu/g coliforms, yeasts and molds and <10,000 cfu/g aerobic plate count (APC)), which is similar to other protein powders generally recognized as safe (GRAS) by the Food and Drug Administration (FDA) [52] and is not a significant source of bacteria. The *Bifidobacterium longum* supbsp. *infantis* (UCD272) culture was provided by Culture Systems Inc. The probiotic dose administered in this study was 20 billion CFU per day, which was based on several previous clinical trials assessing tolerability of *B*. *infantis* in preterm infants (birth weight <1500 grams, gestational age < 33 weeks) at high risk for necrotizing enterocolitis (NEC) [53, 54]. A dose of 4 x 10^9^ CFU twice daily was well-tolerated in these infants with no vomiting, diarrhea, feeding intolerance, or NEC incidence. The full study protocol is available upon request by the corresponding author.

**Table 2:** Composition of liquid bovine colostrum product.

**Oligosaccharides**	**Content (g/L)**
3 and 6 sialyllactose	5.2
3 and 6 sialyllactosamine	4.2
Free sialic acid	1.6
LNnT and other HexNAc	1.8
Trioses	1.0
**Other Nutrients**	**Content (mg/100mL)**
Calcium	58.1
Sodium	53.6
Potassium	118
Calories	23.7
**Specific proteins**	**Content**
Insulin	0 ng/mL
Relaxin	411 pg/mL
Lactoferrin	178.18 mg/mL
TGF-β2	0.232 ng/mL
IGF-1	Not detected

LNnT, Lacto-N-neotetraose; HexNAc, N-acetylglucoseamine or N-acetylgalactoseamine; TGF-β2, tumor growth factor β2; IGF-1, insulin-like growth factor 1

Parents were instructed to store the powdered supplement in the freezer and mix a single dose once daily into a cold food item (usually yogurt, milk, juice or ice cream) as a part of their child’s regular daily diet for a period of 5 weeks. Parents were asked not to change the child’s diet or any other treatments while participating in the study. Any children currently taking medication for GI symptoms (*e*.*g*. laxatives, stool softeners) were asked to temporarily suspend use of these medications a month before participating in the study until study completion. Parents were also asked to complete a dosing diary in order to assess compliance as well as a daily stool log to keep track of their child’s bowel movement frequency and consistency.

At each study visit, a medical examination of the participant was performed by a study physician and GI symptom questionnaires were completed by the parents. Parents were also asked to complete validated questionnaires relevant to behavior. Participant height and weight measurements as well as blood, urine and stool specimen collection were also performed at each visit. All samples were stored at -80°C immediately after collection until the analyses were performed. During the study period, the participants’ well-being was monitored by phone calls from the study staff midway through each study arm. All other observations by parents were also noted. Safety evaluations that included a complete blood count (CBC) analysis, physical examinations and questions about adverse events were carried out by the study physician.

### Questionnaires and logs

GI symptom questionnaires included the CHARGE Gastrointestinal History (GIH) survey [55] and the Questionnaire on Pediatric Gastrointestinal Symptoms-Rome III Version (QPGS-RIII). The GIH includes 10 Likert scale items (0 = never; 1 = rarely; 2 = sometimes; 3 = frequently; 4 = always) for each current gastrointestinal symptom (abdominal pain, gaseousness/bloating, diarrhea, constipation, pain on stooling, vomiting, sensitivity to foods, difficulty swallowing, blood in stool and blood in vomit). “Current” was defined as the past three months. Additionally, the GIH includes four (yes/no) questions asking about the presence of food allergies, diet restrictions, food dislikes and whether any GI diagnosis has ever been given. Finally, open-ended questions asked parents to list: food allergies; reasons for diet/food restrictions; and what GI condition was diagnosed. The QPGS-RIII questionnaire data were prospectively collected and scores were determined by subtracting post-intervention values minus pre-intervention values and then normalizing the difference so that a positive score was indicative of improvement and a negative score was indicative of worsening for questions 2 and 3 only. Behavioral questionnaires utilized for this study included the Aberrant Behavior Checklist (ABC), the Repetitive Behavior Scale-Revised (RBS-R), and the Adaptive Behavior Assessment System–Second Edition (ABAS-II). The stool log contained a 7-point Bristol Stool Scale that parents used to classify stool consistency. The stool log data was collected throughout the study period and frequency and consistency of stools were binned into time periods including the first three days of the study (D123), week 1 of the study (W1), week 5 of the study (W5), and days 8–35 of the study corresponding to week 2 through week 5 (D835). Proportions were calculated based on the number of normal stools (4 on Bristol Scale), hard stools (1 or 2 on Bristol Scale), or soft stools (6 or 7 on Bristol Scale) divided by the total number of recorded stools during the specified time period. Data were received from all study participants so that all eligible participants were included in the analysis (n = 8). The dosing diary included information on the daily timing of supplement administration as well as the amount and type of food mixer used and the amount of supplement-containing food item consumed by the child.

### Fecal DNA extraction

Fecal DNA extractions were performed using the ZR Fecal DNA MiniPrep Kit (Zymo Research Corp., Irvine, CA) according to the manufacturer’s instructions. Cell lysis was carried out using the MP Bio FastPrep24 bead beater at 6.5 m/s for 60 sec, repeated twice followed by 3 min rests in between sets and a final 5 min rest. DNA was eluted in 100 μl elution buffer warmed to 70°C and then stored at -80°C until further use. The microbiota analysis was performed for the BCP only treatment (n = 8) and the combination treatment (n = 7).

### Microbiota analysis

DNA library construction was performed as previously described [56]. Samples were submitted to the UC Davis Genome Center DNA Technologies Core for sequencing on an Illumina MiSeq instrument (Illumina, San Diego, CA). From the resulting sequencing data paired end reads were demultiplexed and merged using Paired-End reAd mergeR (PEAR version 0.9.8 [57]). Quality filtering and downstream data processing was carried out with QIIME software package (version 1.9.1; University of Colorado, Boulder, CO) [58]. Within QIIME, operational taxonomic units were assigned using swarm [59]. Taxonomic classification was based on 97% pairwise identity using the Ribosomal Database Project classifier (Michigan State University, East Lansing, MI) against a representative subset of the Greengenes 16S ribosomal RNA database (gg_13_8 release, Second Genome, South San Francisco, CA) [60, 61].

### Microbial community state analysis

The biom table from the QIIME analysis was used to create a Phyloseq data object [62]. The microbiomes were clustered using the Bray-Curtis distance metric and NMDS ordination method. For this analysis, the Bray-Curtis metric was chosen because it does not account for phylogenetic relatedness and therefore provides maximal separation of communities. Based on a screeplot and eigenvalues, the first 6 dimensions were retained for the NMDS ordination. Next, the number of clusters (K = 4) was determined using the gap statistic. The members of the four clusters were determined using the partition around medoids algorithm, pam, in the R [63] package “cluster” [64].

### Blood collection and cellular assays

Analysis was performed on all available peripheral blood samples (n = 7) collected from each participant in acid-citrate dextrose Vacutainers (BD Biosciences; San Jose, Ca). Each tube was centrifuged at 2100 rpm for 10 minutes, plasma was removed and the remaining blood components were layered onto lymphocyte separation medium (Corning; Manassas, VA), and centrifuged at 1700 rpm for 30 minutes. Peripheral blood monuclear cells (PBMC) from the buffy layer were collected and washed with Hanks balanced salt solution (Corning; Manassas, VA). After isolation, PBMC were allowed to rest overnight in complete media (RPMI 1640 (Invitrogen; Carlsbad, CA) with 10% Fetal Bovine Serum (FBS) (Corning; Manassas, VA), 100 IU/mL penicillin (Invitrogen; Carlsbad, CA) and 100 IU/ml streptomycin (Invitrogen; Carlsbad, CA)). PMBC were then divided and plated in complete media containing 10.6 μM Brefeldin A and 2 μM Monesin (protein transport inhibitor cocktail (ebioscience; San Diego,CA)) or in complete media containing 10.6 μM Brefeldin A, 2 μM Monesin, 81 nM Phorbol 12-Myristate 13-Acetate (PMA) and 1.34 μM Ionomycin (Cell Stimulation Cocktail (plus protein transport inhibitors) (ebioscience; San Diego, CA)) for 4 hrs. Following stimulation, PBMC were washed with PBS (Corning; Manassas, VA), 1 x 10^6^ cells per well were plated for each condition and stained with 100 μL of Live/dead amine dye (LIVE/DEAD Fixable Aqua Dead Cell Strain Kit, for 405 nm excitation (Invitrogen; Carlsbad, CA) for 20 mins in the dark. PBMC were washed with PBS, then reconstituted in 100 μL of PBMC wash (PBS (Corning), FBS (Corning), sodium azide (Sigma-Aldrich; St. Louis, MO)) containing 10% FcR block (Miltenyi Biotec; San Diego, CA). Cells were incubated in the dark for 10 minutes at 4°C, cell surface staining antibody cocktails were added and cells were incubated in the dark for another 30 minutes at 4°C. Following staining cells were washed 3x with PBMC wash and resuspended in 100 μL of 1x Fix/Perm (BD Cytofix/Cytoperm solution; BD Bioscience, San Jose, CA)) and incubated in the dark for 20 minutes at 4°C. Cells were washed 2x with perm wash buffer (BD Perm/Wash buffer (BD bioscience)) and resuspended in 50 μL of perm wash buffer and intracellular antibody cocktails, followed by a 30-minute incubation in the dark at room temperature. Cells were subsequently washed 2 times with perm wash buffer then resuspended in 100 μL 1% paraformaldehyde (Sigma-Aldrich). Cells were stored in the dark at 4°C until analysis. Antibodies were purchased from BioLegend: anti-human CD3 (clone UCHT1)-brilliant Violet 421; anti-human integrin β7 (clone FIB27) PerCP/Cy5.5; anti-human LAP (TGF-β1) (clone TW4-2F8)-APC; anti-human FoxP3 (clone 206D)-PE; anti-human IL-6 (clone MQ2-13A5)-PE. The following antibodies were purchased from eBioscience: anti-human CD8α (clone OKT8)-Alexa Fluor 700; anti-human IL-17A (clone eBio64DEC17)-FITC; anti-human IL-13 (clone 85BRD)-FITC; anti-human CD25 (clone BC96)-Alexa Fluor 488; anti-human IL-10 (clone JES3-9D7)-PE; anti-human TNFα (clone Mab11)-FITC. Anti-human IFNγ (clone B27)-PE was purchased from BD Bioscience.Flow cytometric acquisition was performed on a LSR II flow cytometer (BD Biosciences) using FACSDiva software (BD Biosciences) and 100,000 acquired events were captured for each antibody staining condition. Flow cytometry data was analyzed with Flowjo software (Tree Star, Inc; Ashland, OR). Lymphocytes were gated using forward scatter and side scatter parameters. Dead cells were excluded and live cells were gated for presence of CD3 (T cells).

### Serum, urinary and fecal metabolomics

Analyses were performed on all samples available from participants for serum (n = 7), urine (n = 8), and fecal (BCP only n = 8, combination n = 7) metabolomics. For fecal metabolomics, 400–500 mg of frozen stool was weighed and 10 mM phosphate buffer (1/5 (w/v)) was added to each sample. Samples were vortexed for 2 min at max speed, placed on ice, and the process repeated. Fecal samples were then centrifuged at 5.5k for 15 minutes. The supernatant (850 μL) from each sample was then transferred to a new tube and centrifuged again at 14k for 10 minutes, followed by filtration with a 0.22 μm syringe filter. For urinary and serum metabolomics measurement, samples were prepared by thawing and centrifuging at maximum speed for 40 min for urine and 50 min for serum and 14k rcf to remove particulate matter. All sample supernatants were subsequently filtered through Amicon 3,000 molecular weight (MW) cutoff filters to remove protein and lipid particles. An internal standard (Chenomx Inc., Edmonton, Alberta, Canada) containing 4.8566 mM 3-(trimethylsilyl)-1-propanesulfonic acid-d 6 (DSS-d6) and 0.2% NaN3 in 98% D2O (23 μL) was added to a 207 μL aliquot of each of the sample filtrates. The pH value was adjusted to 6.8 for each sample by adding small volumes of 1 N HCl or NaOH. 180 μL of each sample was transferred into a 3 mm NMR tube, and samples were stored at 4°C until NMR data acquisition (within 24 h of sample preparation). NMR spectra were acquired using the Bruker noesypr1d experiment on a Bruker Avance 600 MHz NMR spectrometer equipped with a SampleJet as previously described [65]. Identification and quantification of metabolites were accomplished using Chenomx NMRSuite 7.6 (Chenomx Inc., Edmonton, Canada) [66]. For each fecal sample, 50 metabolites were detected and quantified including 1,3-dihydroxyacetone, 2-oxocaproate, 3-phenylpropionate, 4-hydroxyphenylacetate, acetate, alanine, arabinose, arginine, aspartate, butyrate, DL-methionine-sulfoxide, ethanol, formate, fumarate, galactose, glucose, glutamate, glycerol, glycine, hypoxanthine, isobutyrate, isoleucine, isovalerate, leucine, lysine, malonate, maltose, methanol, methionine, methylamine, N-acetylglutamate, nicotinate, ornithine, phenylacetate, phenylalanine, proline, propionate, ribose, serine, succinate, taurine, threonine, thymine, tryptophan, tyrosine, uracil, uridine, valerate, valine and p-cresol. For each urine sample, 67 metabolites were detected and quantified including 1,6-anhydro-β-D-glucose, 1-methylnicotinamide, 2-aminobutyrate, 2-furoylglycine, 2-hydroxyisobutyrate, 2-oxoglutarate, 2-oxoisocaproate, 3-aminoisobutyrate, 3-hydroxy-3-methylglutarate, 3-hydroxyisobutyrate, 3-hydroxyisovalerate, 3-hydroxyphenylacetate, 3-indoxylsulfate, 4-hydroxyphenylacetate, 4-hydroxyphenyllactate, acetate, alanine, allantoin, arabinose, ascorbate, asparagine, betaine, choline, citrate, creatine, creatinine, dimethylamine, formate, fructose, fucose, galactose, glucose, glutamine, glycerol, glycine, glycolate, hippurate, histidine, hypoxanthine, isobutyrate, isoleucine, isopropanol, lactate, leucine, mannitol, methylguanidine, methylsuccinate, pantothenate, phenylalanine, propylene glycol, quinolinate, serine, succinate, sucrose, taurine, threonine, trigonelline, trimethylamine N-oxide, tryptophan, tyrosine, urea, valine, xylose, cis-aconitate, sn-glycero-3-phosphocholine, trans-aconitate and π-methylhistidine. For each serum sample, 38 metabolites were detected and quantified including 2-hydroxybutyrate, 2-hydroxyisovalerate, 2-oxoglutarate, 3-hydroxybutyrate, 3-hydroxyisobutyrate, acetate, acetoacetate, acetone, alanine, ascorbate, asparagine, betaine, carnitine, choline, creatine, creatinine, ethanol, formate, glucose, glutamate, glutamine, glycine, isoleucine, lactate, leucine, lysine, mannose, methionine, ornithine, phenylalanine, proline, pyruvate, serine, taurine, threonine, tyrosine, urea and valine.

### Statistical analysis

Statistical differences in scores for all questionnaire data and intracellular cytokine expression pairwise comparisons before and after each study arm were determined by means of Student’s paired sample t-test with p ≤ 0.05 considered significant using R version 3.3.1 statistical software. Analyses were also performed using the Wilcoxon rank-sum test for non-parametric data, with similar results to those from the t-tests. Therefore, results from the t-test analyses are presented in the text and tables, along with the 95% confidence intervals. Statistical differences in metabolite levels were determined by compiling data for both treatment arms and comparing pre-treatment to post-treatment values using the Wilcoxon test for non-parametric data. Graphs were generated using GraphPad Prism version 7.02 software. Microbiota principal coordinate analysis (PCoA) was performed using Emperor software. Statistical differences in abundances of microbial taxa were conducted by adapting statistical methods from RNA-sequencing [67]. Specifically, likelihood ratio tests were conducted using DESeq2 [68]. Metabolomic principal component (PCA) plots were generated using log-transformed data and SIMCA software. Carry over effect was determined for select outcome measures in order to determine if the subjects returned to baseline after the washout period following the first treatment arm using Student’s two sample t-test comparing order of treatment on outcomes. Briefly, subjects were grouped based on order of treatment and within-arm changes for primary outcomes measures was determined for each treatment. A difference in outcome by group was interpreted as an effect of treatment order and thus insufficient wash out (carry over) while lack of significant difference in outcome by group was interpreted as no effect of treatment order and thus sufficient wash out (no carry over).

## Results

### Study participation

Twenty children with a diagnosis of ASD and who had gastrointestinal symptoms were screened for eligibility to participate in the study. Eleven children met initial study criteria, all of which were randomly assigned to receive the combination or BCP supplementation first (Fig 1). Screen failures were due to declined participation (n = 7), unwillingness to stop medications (n = 1) and unwillingness to provide blood samples (n = 1). Two participants exited the study during the first arm due to time constraints (n = 1) and other family medical issues (n = 1). While nine participants completed the study, eight were included in the final analysis. One participant was excluded from the analysis due to a post-trial diagnosis of celiac disease. Of the participants included in the final analysis, four received the combination supplementation first while four received the BCP only supplementation first. Biological samples were successfully collected for most study participants. One participant (208) failed to provide a stool sample after the wash out period so this participant’s microbiota and fecal metabolomics data were not included for the second arm of the study. One participant also failed to provide blood samples due to excessive anxiety and restlessness during the venipuncture procedure so his data was not included in the blood cytokine or metabolomics data. Overall, participant compliance with study protocol was high with an average of 93.9 ± 8.6% (72.5–100%) of the study supplement prescribed reportedly consumed by the study participants over both trial arms based on data obtained from parentally-reported dosing diaries. These data were more accurate than assessing returned supplement as the study supplement was often all mixed into a food item but was incompletely consumed thereafter.

### Participant characteristics

The average age of study participants (Table 1) was 6.8 ± 2.4 years (3.9–10.9 years) with a male to female ratio of 7 to 1. While diagnostic assessments during screening indicated that two participants were eligible for functional constipation, five were eligible for functional diarrhea, and one was eligible for both, several of the participants experienced symptoms of both diarrhea and constipation at some point during the study (n = 4). Only one participant experienced solely constipation symptoms and three participants experienced only diarrhea symptoms based on questionnaire and stool log data. In addition, several participants (n = 5) were enrolled with a functional diagnosis of irritable bowel syndrome as well. Most of the participants (n = 6) experienced pain associated with bowel movements at some point during the study. Most of the study participants (n = 7) experienced gassiness and bloating during the study period as well. No parent reported use of antibiotics during the 3-month period prior to the study onset and many responded that there was no antibiotic use in the prior year. Only one participant was using a laxative at the start of the study and was required to cease use prior to enrollment. The majority of the study participants (n = 7) were reported to be picky eaters by their parents and often consumed self-restricted diets. All study participants reported food/taste sensitivity on the GIH. However, all of the study participants were already regular consumers of dairy products.

### Supplement tolerability and side effects

In general, both the BCP only and the combination treatment (BCP + *B*. *infantis*) were well tolerated in this cohort with no participants needing to withdraw due to adverse events. However, parents of 3 participants reported that their children did not like the taste of the supplement, although all were compliant with study protocol and were able to complete the study. The most commonly reported side effects included increased gassiness (n = 2), stomachache (n = 1), sleep disturbances (n = 1) and lethargy (n = 1) on the BCP only arm and weight gain (n = 2), increased gassiness (n = 2) and hives (n = 1) on the combination treatment arm. However, sleep disturbances, lethargy and hives were deemed not related to treatment by the study physician given other environmental factors. There were no significant changes in subject weight or body mass index (BMI) for either treatment or between treatments (Tables 3 and S1). There was also no carry over effect for changes in microbial taxa, stool frequency, stool consistency, frequency of GI symptoms and ABC scores, such that the outcomes were similar if the participants received the BCP only treatment first compared to receiving the combination treatment first (data not shown). Only one outcome measure was shown to have a significant order effect. Frequency of diarrhea, based on the GIH survey, showed a mean increase in symptoms on the combination arm if they received BCP only first (mean score change = 0.25) and a mean decrease in scores on the combination arm if they received the combination treatment first (mean score change = -1.25) that was statistically significant (p = 0.005, 95% CI [0.635, 2.365]). However, there was no difference based on order for this outcome measure on the BCP only treatment.

**Table 3:** Confidence intervals and p-values for gastrointestinal and behavioral questionnaire data.

Outcome Variable	BCP Only Treatment (mean ± SD)[95% CI] (n = 8)	Combination Treatment (mean ± SD)[95% CI] (n = 8)	Treatment Comparison (mean ± SD)[95% CI] (n = 8)
**Diarrhea Frequency**	**-0.875 ± 0.835**	-0.5 ± 0.926	-0.375 ± 1.506
	**[-1.573, -0.177]**	[-1.274, 0.274]	[-1.634, 0.884]
	**P = 0.021**	P = 0.171	P = 0.504
**Constipation Frequency**	-0.563 ± 1.545	-0.5 ± 0.756	-0.063 ± 1.148
	[-1.855, 0.730]	[-1.132, 0.132]	[-1.022, 0.897]
	P = 0.338	P = 0.104	P = 0.882
**Pain Frequency**	**-0.938 ± 1.084**	**-0.75 ± 0.707**	-0.188 ± 1.067
	**[-1.843, -0.032]**	**[-1.341, -0.159]**	[-1.079, 0.704]
	**P = 0.044**	**P = 0.020**	P = 0.634
**Gas Frequency**	-0.875 ± 1.126	-0.75 ± 1.165	-0.125 ± 1.458
	[-1.816, 0.066]	[-1.724, 0.224]	[-1.344, 1.094]
	P = 0.064	P = 0.111	P = 0.815
**Frequency Normalization**	0.188 ± 0.843	0.625 ± 1.026	-0.438 ± 0.980
	[-0.517, 0.892]	[-0.233, 1.483]	[-1.159, 0.284]
	P = 0.549	P = 0.129	P = 0.195
**Consistency Normalization**	**1.063 ± 1.208**	**0.563 ± 0.500**	0.5 ± 1.195
	**[0.052, 2.073]**	**[0.148, 0.977]**	[-0.421, 1.421]
	**P = 0.042**	**P = 0.015**	P = 0.240
**Weight Change**	0.725 ± 1.491	0.138 ± 2.393	0.588 ± 2.615
	[-0.521, 1.971]	[-1.863, 2.138]	[-1.599, 2.774]
	P = 0.211	P = 0.876	P = 0.545
**BMI Change**	0.15 ± 0.521	-0.263 ± 0.901	0.413 ± 0.913
	[-0.286, 0.586]	[-1.015, 0.490]	[-0.350, 1.175]
	P = 0.442	P = 0.437	P = 0.242
**ABC–Irritability Score Change**	**-6.375 ± 3.998**	-2.125 ± 7.754	-4.25 ± 9.910
	**[-9.717, -3.033]**	[-8.608, 4.358]	[-12.535, 4.035]
	**P = 0.003**	P = 0.464	P = 0.265
**ABC–Lethargy Score Change**	-5.125 ± 6.958	**-2.0 ± 2.390**	-3.125 ± 8.254
	[-10.942, 0.692]	**[-3.998, -0.002]**	[-10.025, 3.775]
	P = 0.076	**P = 0.0499**	P = 0.320
**ABC–Stereotypy Score Change**	**-3.0 ± 2.204**	-0.875 ± 1.642	**-2.125 ± 2.295**
	**[-4.843, -1.158]**	[-2.248, 0.498]	**[-4.044, -0.206]**
	**P = 0.006**	P = 0.176	**P = 0.034**
**ABC–Hyperactivity Score Change**	**-6.25 ± 4.743**	-2.5 ± 5.014	-3.75 ± 6.251
	**[-10.216, -2.284]**	[-6.692, 1.692]	[-8.976, 1.476]
	**P = 0.007**	P = 0.201	P = 0.134
**ABC–Total Score Change**	**-21.5 ± 15.556**	-6.75 ± 16.237	-14.75 ± 24.668
	**[-34.505, -8.495]**	[-20.325, 6.825]	[-35.373, 5.873]
	**P = 0.006**	P = 0.278	P = 0.135

Bold = statistically significant (p<0.05)

### Gastrointestinal symptoms

**Fig 4:**
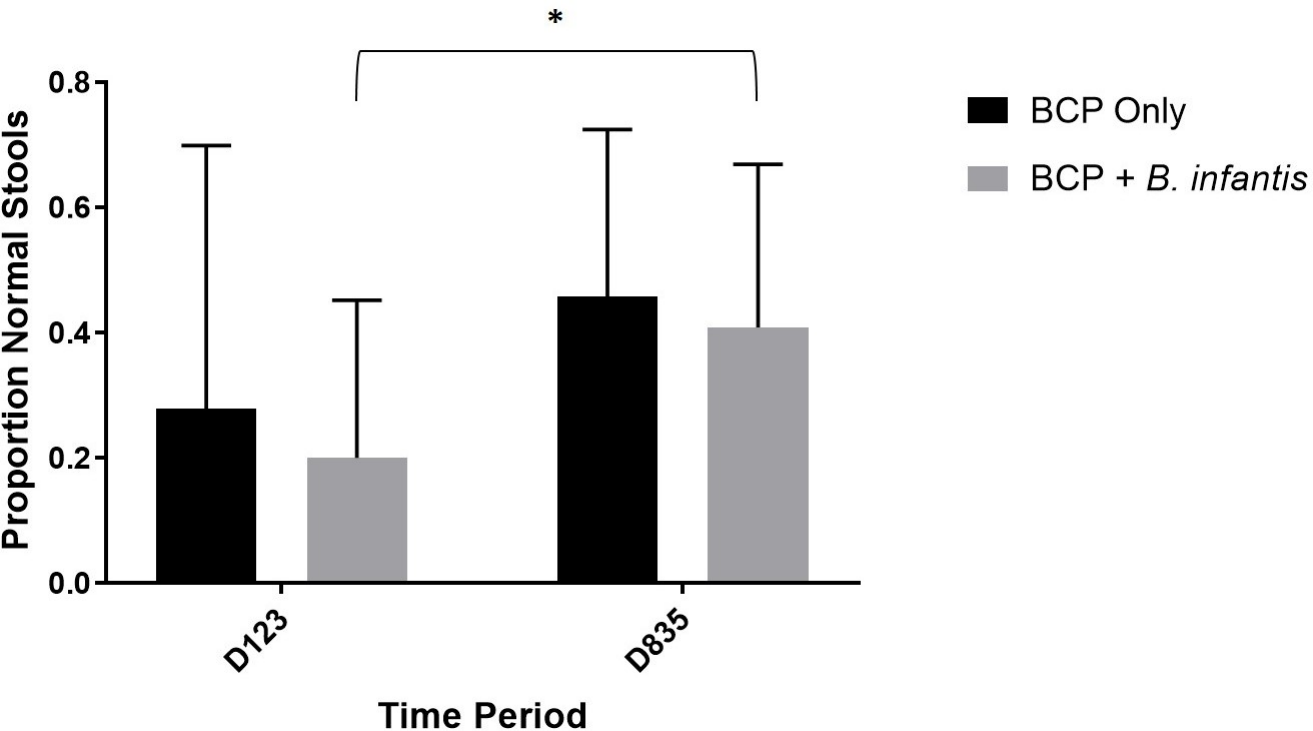
Proportion of normal stools with treatment. Mean ± SD proportion of total recorded stools that were of normal consistency (4 on Bristol Stool Scale), based on stool log data (n = 8 for each group). Significant differences in means (p<0.05) are denoted by an asterisk. D123, days 1,2 and 3 of the study period (baseline); D835, days 8 through 35 of the study period.

Based on overall results from both QPGS-RIII and GIH questionnaire data and parental reporting, 87.5% (7/8) of participants exhibited some improvement in GI symptoms while on the BCP only arm and 100% (8/8) of participants exhibited some improvement in GI symptoms while on the combination treatment arm. When parents were asked on which arm they felt their child had shown greater global improvement in GI symptoms, 75% (6/8) reported greater improvement on the BCP only arm while 25% (2/8) reported greater improvement on the combination treatment arm. Based on parentally reported stool log data, there was a significant increase in the percentage of stools that were of normal consistency (4 on Bristol Stool Scale) from a low proportion at baseline (near 20%) to nearly double that proportion (around 40%) after the intervention period for the combination treatment arm (p = 0.047) (Fig 4)(Tables 4 and S2). Concurrent with increases in the proportion of normal stools, the proportion of hard and soft stools tended to decrease with treatment (Figures A and B in S1 Fig). There was also a reduction in the proportion of soft stools from week 1 versus weeks 2–5 (p = 0.047) for the combination treatment group (Table 4). There was a trend toward significant decrease in hard stool for week 1 versus week 5 (p = 0.059) and week 1 versus weeks 2–5 (p = 0.082) for the BCP only treatment (Table 4). In 7 of the 8 children, parents reported a return of GI symptoms after the supplement was discontinued.

**Table 4:** Descriptive statistics for stool consistency from stool log data.

Time Periods	Proportion Normal Consistency (n = 8)			Proportion Hard Consistency (n = 8)			Proportion Soft Consistency (n = 8)		
	BCP Only Treatment	Combination Treatment	Treatment Comparison	BCP Only Treatment	Combination Treatment	Treatment Comparison	BCP Only Treatment	Combination Treatment	Treatment Comparison
**D123 vs D835 (Mean Diff [95%CI])**	0.13	**0.2**	0.16	-0.13	-0.055	-0.08	-0.07	-0.23	0.16
	[-0.202, 0.453]	**[0.004, 0.387]**	[-0.002, 0.323]	[-0.440, 0.178]	[-0.302, 0.192]	[-0.333, 0.180]	[-0.455, 0.318]	[-0.567, 0.118]	[-0.465, 0.777]
	P = 0.385	**P = 0.047**	P = 0.052	P = 0.338	P = 0.604	P = 0.495	P = 0.680	P = 0.160	P = 0.561
**W1 vs D835 (Mean Diff [95%CI])**	0.153,	0.08	0.07	-0.26	-0.032	-0.22	-5.7x10^-5^	**-0.11**	0.11
	[-0.155, 0.461]	[-0.018, 0.185]	[-0.293, 0.433]	[-0.555, 0.043]	[-0.224, 0.160]	[-0.559, 0.111]	[-0.185, 0.185]	**[-0.225, -0.002]**	[-0.148, 0.375]
	P = 0.279	P = 0.094	P = 0.663	P = 0.082	P = 0.702	P = 0.159	P = 0.999	**P = 0.047**	P = 0.338
**W1 vs W5 (Mean Diff [95%CI])**	0.15	0.06	0.09	-0.2	-0.041	-0.16	-0.01	-0.13	0.12
	[-0.126, 0.426]	[-0.137, 0.250]	[-0.220, 0.408]	[-0.408, 0.009]	[-0.120, 0.038]	[-0.373, 0.057]	[-0.212, 0.199]	[-0.268, 0.006]	[-0.107, 0.355]
	P = 0.239	P = 0.504	P = 0.530	P = 0.059	P = 0.25	P = 0.136	P = 0.939	P = 0.058	P = 0.266
**D123 vs W5 (Mean Diff [95%CI])**	0.16	0.16	0	-0.08	-0.059	-0.02	-0.08	-0.23	0.15
	[-0.154, 0.473]	[-0.086, 0.399]	[-0.400, 0.406]	[-0.253, 0.091]	[-0.164, 0.046]	[-0.169, 0.126]	[-0.462, 0.310]	[-0.579, 0.127]	[-0.495, 0.795]
	P = 0.259	P = 0.165	P = 0.987	P = 0.294	P = 0.217	P = 0.730	P = 0.646	P = 0.168	P = 0.591

D123, days 1–3 of study arm; D835, days 8–35 of study arm; W1, days 1–7 of study arm; W5, days 28–35 of study arm

Bold = statistically significant (p<0.05)

**Fig 5:**
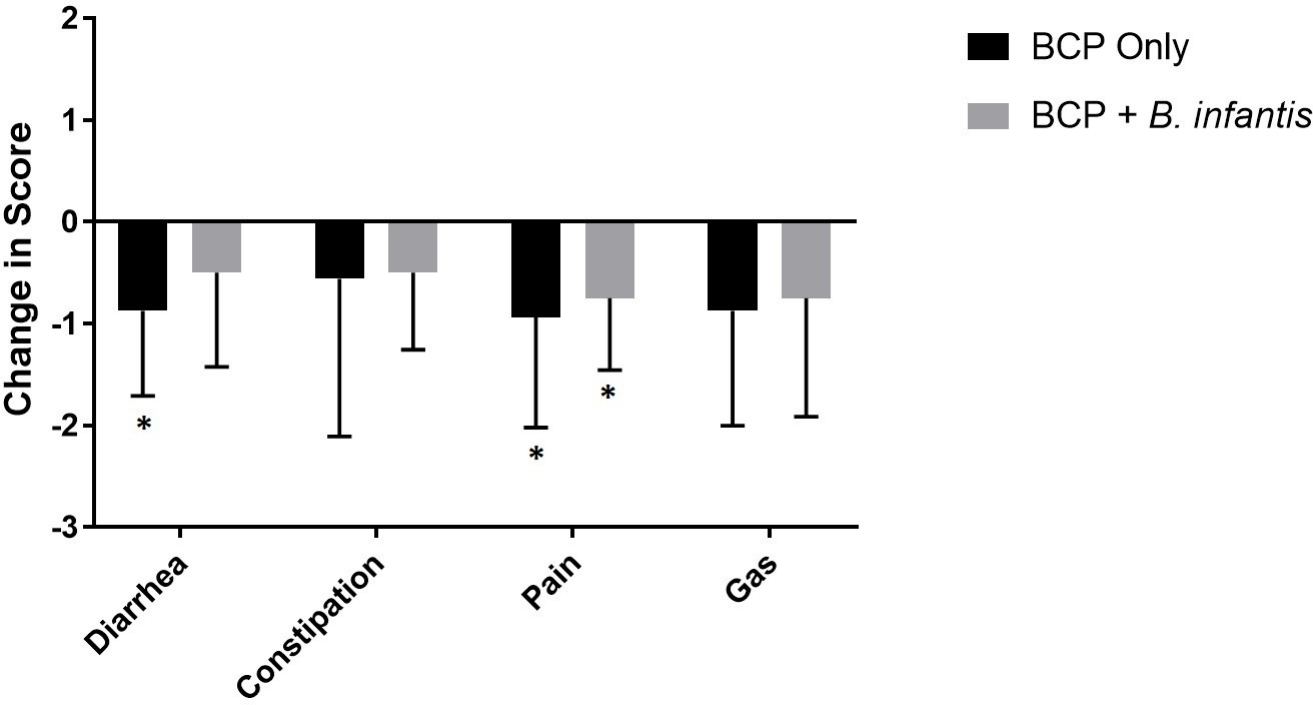
Change in gastrointestinal symptom frequency with treatment. Mean ± SD change in parental reported frequency of gastrointestinal symptom after treatment based on Likert-type scale where 0 = symptom never occurs, 1 = symptom rarely occurs, 2 = symptom sometimes occurs, 3 = symptom often occurs or 4 = symptom always occurs (n = 7 for diarrhea, n = 5 for constipation, n = 5 for pain and n = 6 for gas per group). Means that are significantly different from 0 (p<0.05), indicating significant improvement, are denoted by an asterisk.

Based on the GIH survey, a reduction in certain GI symptoms was observed, specifically pain with stooling, frequency of diarrhea, and consistency. There was a reduction in frequency of pain associated with bowel movements for both the BCP only (-0.938, 95% CI [-1.843, -0.032], p = 0.044) and the combination treatment (-0.75, 95% CI [-1.341, -0.159], p = 0.020) groups (Tables 3 and S1 and Fig 5). There was also a reduction in frequency of diarrhea in the BCP only group (p = 0.021) (Tables 3 and S1 and Fig 5). While changes in frequency of normal stools were not significant for either group, changes in stool consistency were significant for both the BCP only (p = 0.042) and combination treatment (p = 0.015)(Tables 3 and S1). In addition, nearly all study participants were reported to be picky eaters at the start of the study (87.5%; 7/8). Of those participants, 43% (3/7) had increased appetite and consumption of novel foods on the BCP only treatment, while 14% (1/7) had increased appetite and consumption of novel foods on the combination treatment arm. The foods most commonly reported to increase in consumption were fruits and meat.

### Behavioral assessments

**Fig 6:**
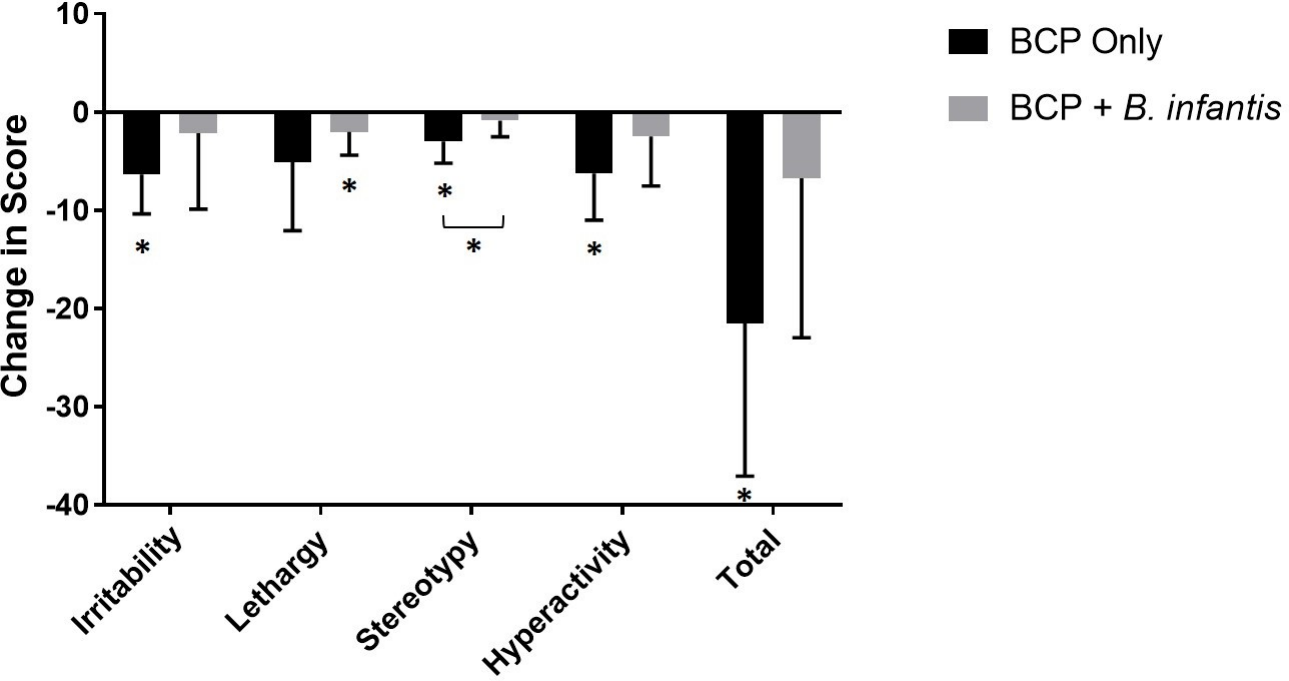
Change in ABC score with treatment. Mean ± SD of change in scores on the Aberrant Behavior Checklist (ABC) based on parental report. Data is presented by subscale as well as total scores (n = 8 per group). Mean differences that are statistically different from 0 (p<0.05), indicating significant improvement, are denoted by an asterisk.

No differences in adaptive behaviors (*e*.*g*. ability for self-care) were observed based on the ABAS-II questionnaire or repetitive behaviors based on the RBS-R (data not shown). A significant reduction in certain aberrant behaviors was found based on the ABC questionnaire data during the BCP only treatment (Fig 6). In the BCP only group, there was a significant reduction in irritability (-6.375, 95% CI [-9.717, -3.033], p = 0.003), stereotypy (-3.0, 95% CI [-4.843, -1.158], p = 0.006), hyperactivity (-6.25, 95% CI [-10.216, -2.284], p = 0.007) and total scores (-21.5, 95% CI [-34.505, -8.495], p = 0.006), along with a trend toward significant reduction in lethargy (p = 0.076; Tables 3 and S1). The combination treatment demonstrated a significant reduction only in lethargy (-2.0, 95% CI [-3.998, -0.002], p = 0.0499). While there were no significant differences in most scores between treatment groups, there was a significant improvement in stereotypy in the BCP only group compared to the combined treatment group (p = 0.034) (Tables 3 and S1).

### Fecal microbial composition

**Fig 7:**
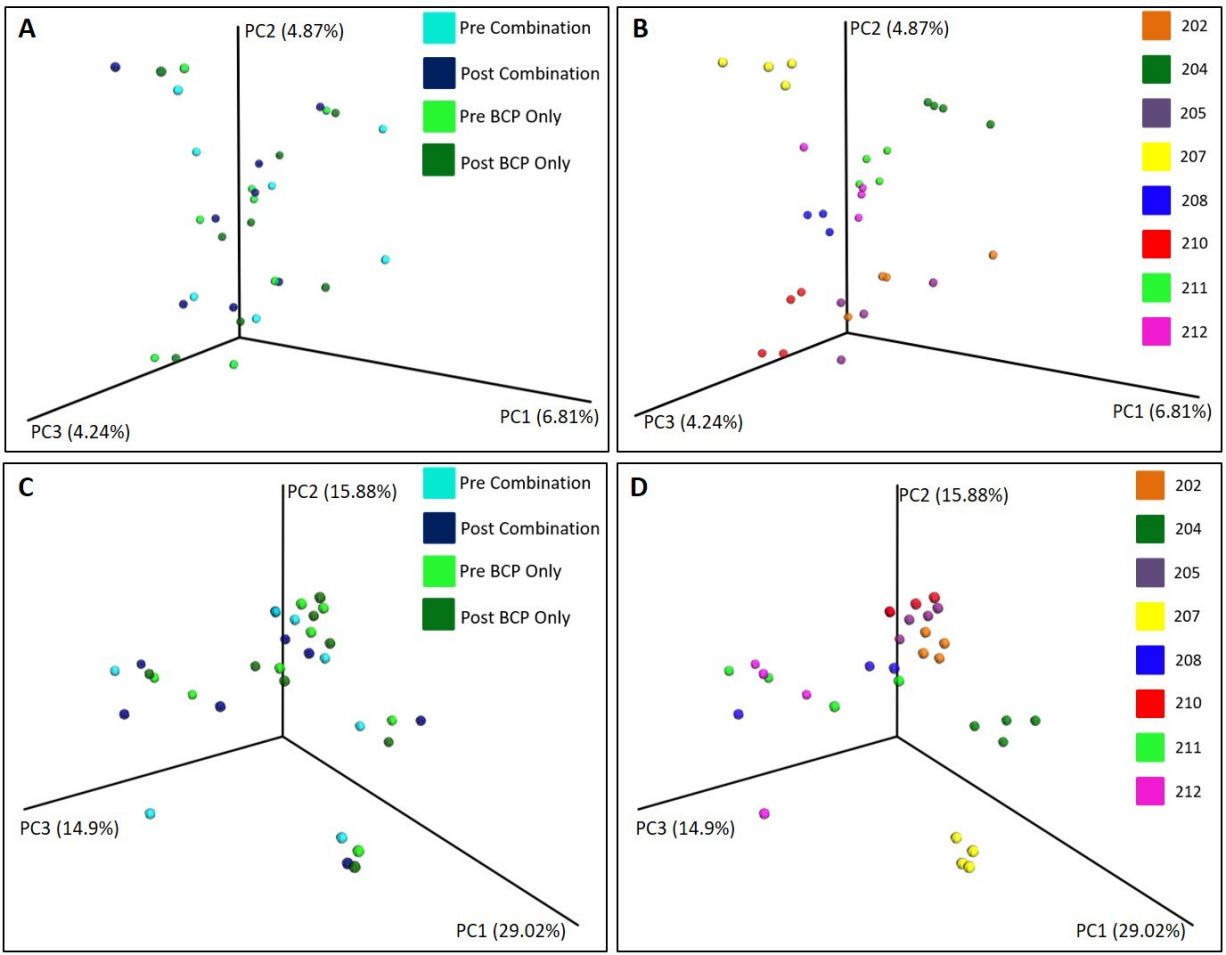
Changes in fecal microbial composition with treatment. UniFrac principal coordinate analysis (PCoA) plots of 16S sequencing data for (A) unweighted data by treatment group, (B) unweighted data by participant, (C) weighted data by treatment group and (D) weighted data by participant; numbers (e.g. 204, 212) represent participant IDs.

UniFrac principal coordinate analysis (PCoA) plots of 16S sequencing data were produced using both weighted and unweighted relative abundance data (Fig 7). Both plots reveal a general lack of global changes to the fecal microbiome with either treatment (Fig 7A and 7C) as evidenced by lack of clustering or clear shifts with treatment. It appears that many individuals’ fecal microbiome profiles clustered together, indicating that some participants’ microbiota were most similar to themselves throughout the study period (Fig 7B and 7D).

**Fig 8:**
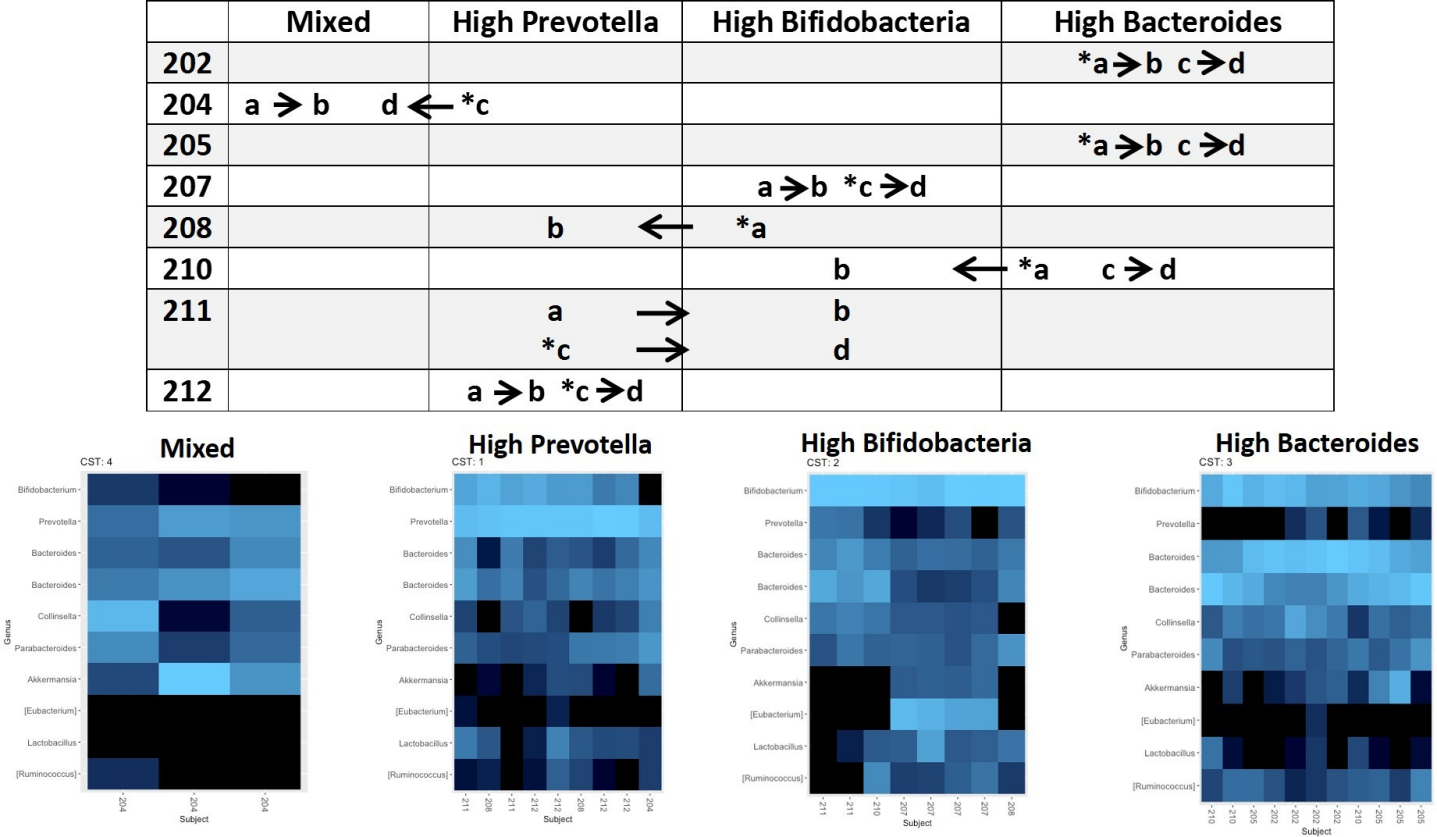
Microbial community state analysis. The bottom half of the figure shows four heatmaps corresponding to each of the four enterotypes identified in the study with the abundances of each bacterial taxon displayed by color (bright blue = more abundant; black = no/low abundance). The top half of the figure shows a chart indicating the enterotype of each participant during the various phases of the study. Arrows show the direction of transitions between enterotypes. The numbers (e.g. 204, 212) represent participant IDs to track transitions of individual participant microbiomes between enterotypes. The treatment period is indicated by a letter (a = pre BCP only treatment, b = post BCP only treatment, c = pre combination treatment, d = post combination treatment). The first arm that the participant was enrolled in is denoted by an asterisk.

It is possible to classify microbiomes by groups of microbial communities or enterotypes, each of which is often dominated by a particular organism [69, 70]. It has also been shown that gut microbiome enterotypes can be affected by diet and can change within the same individual [71, 72]. To further explore how enterotypes differed between individuals and how they were affected by supplementation, a community state analysis was conducted (see Methods). This unsupervised exploration of the data yielded a set of 4 enterotypes (Fig 8): a community high in *Prevotella* (9 samples), a community high in *Bifidobacterium* (8 samples), a community high in *Bacteroides* (11 samples), and a “mixed” community (3 samples). The “mixed” enterotype refers to communities that did not fall into the other three enterotypes (high *Prevotella*, high *Bifidobacterium*, or high *Bacteroides*). All three samples in the “mixed” community were from the same participant (204) with one of the samples higher in *Akkermansia*, one sample higher in *Collinsela*, and one sample moderately high in both *Prevotella* and *Bacteroides*. Consistent with the clustering in the PCoA plots (Fig 7), the microbiome of half of the participants (202, 205, 207, and 212) did not change enterotype throughout the study (Fig 8). The effects of the treatments were inconsistent for the other participants. Participant 204 began the study with a high *Prevotella* enterotype, moved to the mixed enterotype with the first treatment (combination) and remained in that state for the duration of the trial. Participant 208 began the study with a high *Bifidobacterium* enterotype, and moved to the high *Prevotella* enterotype with the BCP only treatment. Participant 210 began the study with a high *Bacteroides* enterotype, moved to the high *Bifidobacterium* enterotype with the BCP only treatment, returned to the high *Bacteroides* enterotype after the washout period and remained in that enterotype with the combination treatment. Participant 211 was in the high *Prevotella* enterotype at the start of each treatment period; both the BCP only and combination treatment moved this participant into the high *Bifidobacterium* enterotype. In summary, the treatments appeared to have either no effect or an inconsistent effect on enterotype.

Next, individual taxa were examined for differential abundance between treatment groups. Taxa were aggregated at the genus level (80 present). Conducting a likelihood ratio test to compare the full study design inclusive of the Treatment and Subject:Treatment interaction terms and a reduced model without these terms, there were no differentially abundant taxa. This suggests there was no treatment effect on any particular genera.

### Metabolomic changes

**Fig 9:**
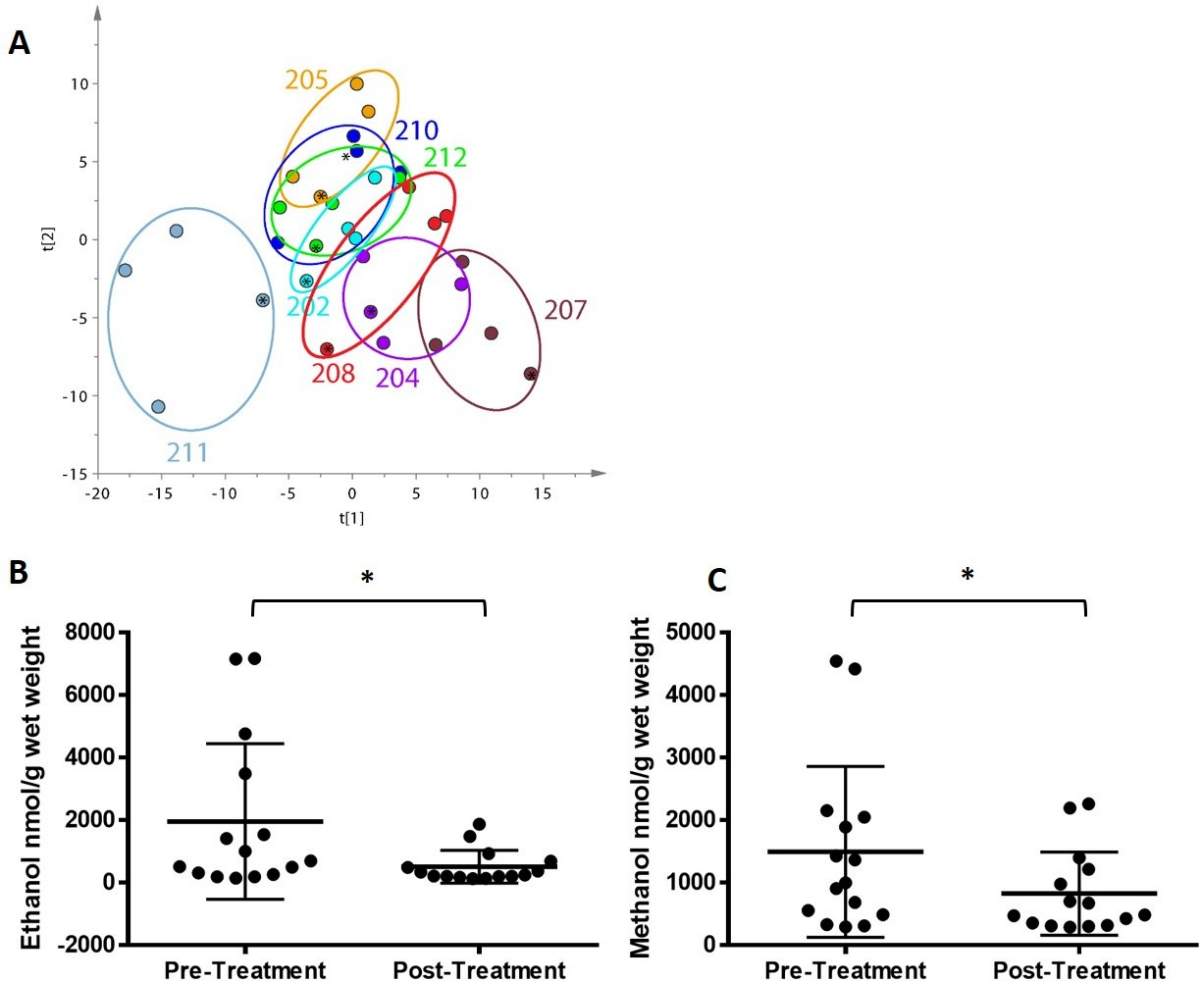
Changes in fecal, urinary and serum metabolomes with treatment. (A) Principal component analysis (PCA) plot of global fecal, urinary and plasma metabolomes changes by participant where numbers (e.g. 204, 212) represent participant IDs and the final metabolome (4th visit) is denoted by an asterisk, (B) mean ± SD fecal ethanol concentrations (n = 15 per group), (C) mean ± SD fecal methanol concentrations (n = 15). Significant differences in mean denoted by an asterisk (p<0.05).

No global changes in fecal, urinary or serum metabolite profiles based on treatment were observed (Fig 9A) and individuals tended to cluster with themselves rather than based on treatment. However, several participants displayed high initial fecal ethanol and methanol concentrations (Fig 9B and 9C). Both ethanol and methanol levels were significantly reduced (p = 0.0256 for both) after treatment. There were no significant differences between treatments in terms of initial ethanol and methanol levels or change in ethanol and methanol levels. There were no significant changes observed for any of the urine or serum metabolites (data not shown).

### Cellular cytokine responses

**Fig 10:**
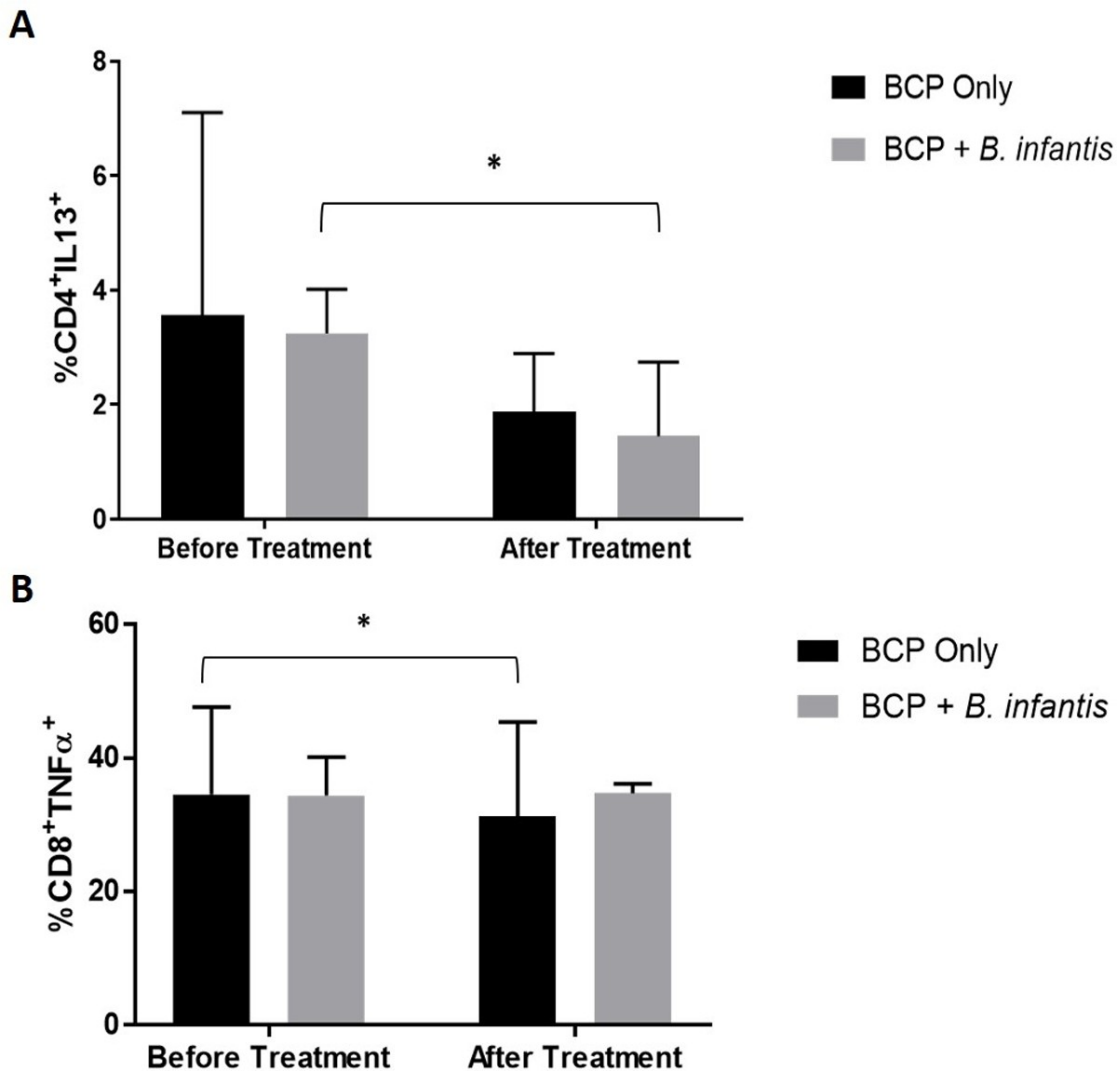
Changes in cellular cytokine production with treatment. Median and interquartile range of percentage of stimulated lymphocytes expressing intracellular cytokines before and after treatment (n = 7 per group). (A) Percentage of stimulated CD4^+^ cells producing intracellular IL-13, and (B) percentage of stimulated CD8^+^ cells producing TNF-α, before and after treatment. Significant differences in percentage before and after treatment (p<0.05) are denoted by an asterisk.

Overall, there was a reduction in intracellular expression of certain cytokines by both CD4^+^ and CD8^+^ T cells. In stimulated cells, the frequency of CD4^+^/IL-13^+^ T cells was significantly lower after combination treatment (95% CI [-2.073, -0.524], p = 0.006) (Fig 10A). There was also a significant reduction in the frequency of CD8^+^/TNF-α^+^ T cells with the BCP only treatment (-7.9%, 95% CI [-14.375, -1.425], p = 0.024) (Fig 10B). We found no significant differences in the frequency of CD4^+^ or CD8^+^ T cells expressing IFN-γ, IL-17, or IL-6 (data not shown). We also found no evidence of carry-over effect with these outcome measures.

## Discussion

A substantial number of children with ASD experience chronic gastrointestinal symptoms including constipation, diarrhea, pain associated with bowel movements and gassiness/bloating [6]. The incidence of these symptoms may be due in part to the presence of an abnormal microbiota as well as aberrant immune function in the GI tracts of these children [11, 15, 18]. Early antibiotic exposure, due in part to this population’s increased incidence of ear infections [73], is also associated with ASD [74] and perturbs the gut microbiota [9], leading to dysbiosis and gastrointestinal problems. The probiotic organism *Bifidobacterium longum* subsp *infantis* has been shown to improve gut barrier integrity [33] and reduce expression of inflammatory genes in intestinal epithelial cells [34]. While bovine colostrum not only contains a limited amount of milk oligosaccharides that may promote the growth of this particular bacteria [35, 36], it also contains an abundance of immune proteins, including immunoglobulins, lactoferrin and a range of cytokines, that can further modulate the microbiota [40] and the immune system [40–42]. In order to assess tolerability and gut microbial changes associated with administration of these supplements, we conducted a pilot study with a crossover design in which children with ASD and chronic GI symptoms were supplemented for five weeks with BCP only and the combination BCP + *B*. *infantis* for another five weeks with a two week wash out period in between. There is a need for carefully designed trials of probiotic/colostrum supplementation in children with ASD and GI symptoms to guide parents and families on safety and tolerability, as studies such as this one are an important first step to inform a future study that can better provide conclusions regarding efficacy. Both study supplements were found to be well-tolerated in our cohort with participants experiencing limited and mild adverse events on both treatment arms. Many children with ASD have sensory issues and restricted eating patterns; the ability to obtain high compliance in supplement administration in this population with minimal drop-out is another highlight of this study. However, it is important to note that while the study supplement appeared to be well tolerated in the current cohort, many children with ASD report sensitivity to dairy products and thus this supplement may not be appropriate for all children with ASD and GI symptoms. The most common adverse events were gassiness and weight gain (a positive side effect). Any reported gassiness occurred at the onset of supplementation and then diminished shortly thereafter with a subsequent reduction in gassiness below baseline (Fig 5). This side effect has been reported in other studies of prebiotic supplementation as well [75]. While not statistically significant, weight gain was also experienced by two participants during the study. However, this effect may actually be beneficial considering that these same participants had low initial BMI. Parents of these children reported a chronic low BMI and inability to gain weight over the span of years, likely due to self-restricted diet and low appetite in these participants. In addition, some parents also reported an increased appetite and willingness to consume novel foods, mostly fruit and meat, by their child during the study. This increased appetite and consumption of novel foods may be due to reduced abdominal pain or improved stool evacuation and might partially explain the weight gain experienced by some of these children.

All study participants experienced reduction in at least one gastrointestinal symptom on at least one treatment arm of the study, if not both. Based on overall impressions of the parents, 75% (6/8) reported greater improvement on the BCP only arm while 25% (2/8) reported greater improvement on the combination treatment arm, which is in agreement with GIH and QPGS-RIII data. This finding is important and likely reliable as all participants and parents were blinded as to the order of treatment and there was no apparent difference in taste or texture between the supplements. Based on stool log data, the proportion of stools with normal consistency was low at the start of both treatments (Fig 4). This proportion increased to nearly half at the end of the combination treatment (p = 0.047). Concurrent with increases in the proportion of normal stools, there was a trend toward a decrease in the proportion of hard and soft stools with treatment (Figures A and B in S1 Fig). Based on GIH survey data, there was a significant decrease in diarrhea incidence during the BCP only treatment (p = 0.021, Fig 5) and a significant decrease in pain during both treatment arms (Fig 5). There were no significant differences between treatment groups for any of the GI symptoms. Anecdotally, all parents thought their child benefitted from supplementation in regard to reduction in global chronic gastrointestinal symptoms that could not be ameliorated by various other treatment options. In addition, many parents inquired about the availability of similar commercially-available supplements in order to continue administration to their child. The BCP product was available in liquid (but not powder) form commercially. While the specific probiotic we used in this study was not commercially available, parents were interested in finding any similar strains of bifidobacteria that they could access immediately. While it appears that the study supplement was well-tolerated by the current cohort, a larger study is required in order to be able establish true efficacy of the study supplement in ameliorating GI symptoms in this population.

In order to assess tolerability further, changes in adaptive, repetitive and aberrant behaviors with supplementation were also assessed. We found significant reduction of certain aberrant behaviors, including irritability, lethargy, stereotypy and hyperactivity, with supplementation (Fig 6), mostly in the BCP only group, which is in agreement with improvement in GI symptoms. The absence of a worsening of adaptive behaviors provides evidence that both study supplements were well-tolerated in this cohort. Reductions in aberrant behaviors may be related to decreases in gastrointestinal discomfort associated with supplementation.

The absence of global changes in the gut microbiota of the study participants with treatment (Figs 7 and 8) indicates that changes in GI function are likely unrelated to major changes in existing gut bacterial taxa. There were also no changes in specific bacterial taxa that haven been shown to differ between children with ASD and typically developing children including *Clostridia* and *Desulfovibrio*. *Akkermansia* species, including *Akkermansia muciniphila*, have been found to be reduced in children with ASD compared to typically developing children [5]. Since *A*. *muciniphila* is a mucus-degrading bacterium, the authors speculated that low levels could indicate a thinner mucus layer in children with ASD and thus insufficient substrate to support the high numbers found in healthy children. However, other studies have shown that *Akkermansia* levels are higher in children with ASD compared to typically developing children, although species-level composition was not examined [13]. One participant in our study (204) displayed high levels of *Akkermansia* after the BCP only treatment as part of the “mixed” enterotype (Fig 9). While species-level composition was not determined for this analysis, higher levels of mucus-degrading bacteria may suggest improvement in GI barrier function in this subject. This particular subject demonstrated an improvement in GI function including an increased proportion of normal stools, reduced pain and gassiness as well as a decrease in CD4^+^IL-13^+^ and CD4^+^IL-17^+^ T cells. However, it is clear that the gut microbiota of this individual was unstable and so results must be interpreted with caution. Substantial heterogeneity in microbial communities was observed between participants. Therefore, the lack of or inconsistent treatment effect (Figs 7 and 8) may be in part because the participants began the study in different microbial community states. Our youngest study participant was 3 years of age and the gut microbiome is stable and adult-like at this age [76]. Further, microbial profiles may not return to baseline after a wash-out period, as occurred with Participant 204. While it may be impossible to “reset” the microbiota, repeating the interventions in the same participant and inclusion of placebo arms could help ameliorate this problem. The absence of an increase in *Bifidobacterium* at any taxonomic level might indicate that decreases in non-*B*. *infantis Bifidobacterium* are occurring concurrently with increases in *B*. *infantis* and our analysis is not specific enough to quantify these changes. It is also possible that *B*. *infantis* did not colonize at a detectable level. Indeed, a recent systematic review of randomized controlled trials of probiotic supplementation in healthy adults showed that numerous probiotics show beneficial health effects without concurrent changes in fecal microbial composition, including α-diversity, richness and evenness, based on high-throughput molecular approaches [77]. It has been hypothesized that the molecular mechanisms behind the beneficial effects of probiotic supplementation lie not in the ability of the bacteria to colonize and change the overall composition of the microbiota but rather in the stabilization of the microbial communities already present, making them less susceptible to perturbations from stressors such as antibiotics, poor diet, and psychological stress [78]. Finally, it is also possible that lack of changes to the microbiota community status indicates lack of efficacy of the supplement and the reduction in certain GI symptoms may be due to other factors, such as a placebo effect.

We found no global changes in fecal, urinary or serum metabolite profile with either treatment and individuals tending to cluster with themselves rather than treatment (Fig 9). The fecal and urinary metabolomes of each individual tended to cluster together more tightly than the plasma metabolome. Data from both study arms was combined for individual metabolite analysis since few significant differences were observed between treatments. Interestingly, we did find high initial fecal ethanol and methanol levels that were subsequently reduced after treatment (Fig 9B and 9C). This finding may explain why GI symptom improvement was observed in the absence of changes in bacterial taxa. There is some evidence to suggest that yeast overgrowth in the gut, usually *Candida* or *Saccharomyces* species, can cause an accumulation of ethanol [79, 80]. Recent studies have shown that children with ASD test positive for anti-*Candida* immunoglobulins at a higher rate compared to typically developing children [81]. In addition, GI perturbations were found in 47% of the ASD children with positive test results compared to only 25% of typically developing children. High ethanol levels have also been found in the blood in patients with extreme cases of Crohn’s disease [82] and short gut [83, 84]. While we found no evidence of high ethanol levels in the blood of our participants, high fecal levels could be indicative of yeast overgrowth that is reduced by treatment. Indeed, the prevalence of gastrointestinal yeast infections in children with ASD and GI symptoms has been documented [85]. We did not see any changes in particular metabolites that have been found to differ between children with ASD and typically developing children such us p-cresol, propionic acid or other short chain fatty acids. Lack of global changes in metabolite profile may be due to small sample size or could indicate lack of efficacy of the study supplement so that reduction in GI symptoms may be due to other factors, such as a placebo effect.

We observed significant changes in intracellular cytokine expression in stimulated peripheral blood mononuclear cells (PBMC) after treatment, specifically CD3^+^ T cells. The combination treatment resulted in a significant reduction in the percentage of helper T lymphocytes (CD4^+^ T cells) expressing IL-13 (Fig 10A). This cytokine is important in allergic responses and may be important in GI pathology as many children with ASD and GI symptoms report food allergy. Elevated production of the T_H_2 cytokine IL-13 in children with ASD had been reported [86]. Therefore, reduction in production of this cytokine corroborates our findings of improved GI function and symptom reduction with both treatments. Importantly, there was a significant reduction in the frequency of CD8^+^ T cells expressing TNF-α after BCP only treatment suggesting pro-inflammatory signals are decreased (Fig 10B). The reduction in inflammatory cytotoxic T lymphocytes is concordant with clinical outcomes and suggests that bovine colostrum may have helped to improve GI function through reduction of expression of pro-inflammatory cytokines in the gut. Our results are in agreement with previous findings of elevated TNF-α expression in children with ASD and GI symptoms [87] compared to non-inflamed controls that was similar to participants with Crohn’s disease [22]. It is also interesting to note that several studies have demonstrated increased TNF-α production in PBMCs isolated from children with ASD in response to certain dietary proteins, including bovine milk proteins [88, 89]. Our findings do not support this preliminary *in vitro* data and suggest that consumption of raw bovine colostrum product may be effective at reducing pro-inflammatory cytokine production in the gut.

Surprisingly, there appears to be a trend toward greater reduction in GI symptoms, aberrant behavior and immune imbalance with the BCP only treatment compared to the BCP + *B*. *infantis* combination treatment. This effect may be due to several factors. Our first hypothesis is that the BCP may be promoting the growth of bacteria other than *B*. *infantis* and that in the presence of the probiotic, this alternative bacterial taxon is not able to colonize. Another hypothesis involves the interaction of the probiotic with the components of the BCP. Bovine colostrum is generally high in immune factors [90] that are heavily glycosylated [91]. These glycans have been shown to protect immune proteins from gastrointestinal digestion so that they can retain bioactivity in the gut [37, 38]. However, *B*. *infantis* is able to produce extracellular glycosidases that allow it to cleave and consume these glycan moieties [45, 92]. Therefore, in the absence of the probiotic, the proteins maintain their glycosylation status to exert beneficial immune balancing activity in the gut. On the other hand, in the presence of the probiotic, these bacteria cleave and subsequently consume these protective glycans. Naked immune proteins become susceptible to digestion and no longer retain biological activity in the gut. Unfortunately, our study was not designed to be able to answer this question. The trend toward greater symptom reduction with BCP only treatment was marginal and it is difficult to conclude that the BCP supplement demonstrated greater symptom reduction than the combination treatment due to high variability in response of our participants.

There are several limitations to the current study. The first is the lack of a clear control group receiving a placebo. It is certainly possible that parents and children may have reported improvements due to the placebo effect related to the expectation of a beneficial effect from receiving a therapy [93]. This has resulted in the inability to reproduce results of some uncontrolled trials in placebo-controlled trials. We have partially addressed this limitation by designing a cross-over study where each participant was his own control. It is also important to note that all of these families had tried numerous medical interventions to alleviate their child’s GI symptoms and many of them were skeptical as to the efficacy of the study treatment as well. While improvements in gastrointestinal symptoms may also be due to simple growth and maturation, the chronic nature of these symptoms and the report of return of symptoms upon post-study follow up with parents suggest that this may not be the case. Moreover, our study duration was short (5 weeks on each arm), and we would not expect to see much growth and maturation occurring in such a limited time. Similarly, all of our participants were receiving standard of care treatments for ASD, and there were no changes in these services during the course of the study. While these interventions can also be related to changes in behavior, they are less likely to do so over the short time period of our study.

Another limitation relates to the distribution of GI symptoms in our ASD group, with a greater percentage of our sample experiencing diarrhea rather than constipation (2:1 ratio). This is congruent with some studies of GI symptoms in ASD, with diarrhea occurring 1.5–2 times more than constipation [9, 94, 95] However, others report the opposite, with constipation reported 2–3 times more frequently than diarrhea [96, 97] Based on this review, our sample may not be representative of the larger ASD population, and this is an important consideration when interpreting our findings. There is a wide range in the reported prevalence of GI conditions in ASD, ranging from 9–70% [6], and estimating the true prevalence from existing studies is difficult due to referral and ascertainment bias [6]. The American Academy of Pediatrics has identified the need for prospective, multi-center studies using validated instruments for the ASD population in order to address this issue.

We also recognize the lack of a probiotic only arm that would have been useful in parsing out specific treatment effects. Our rationale for lack of such a group was to maintain blinding to treatment that we determined to be of great importance. The volume of BCP administered was such that a matched volume of a probiotic only treatment was not feasible. Therefore, the only way to keep participants blinded was to administer the BCP on both arms of the study. We also recognize the lack of a control cohort of healthy children (both non-ASD and asymptomatic in terms of GI function) may interfere with our ability to see time-related changes of the microbiome that occur in a healthy children population that is gender and age matched. The use of a cross-over design, where each participant serves as their own control, partially addresses this flaw as well. In addition, the abundance of outcomes based on parental reporting is another limitation to this study, but was also necessary due to the lack of more objective measures of gastrointestinal symptoms and behavior changes. This limitation is of particular concern in studies of children with ASD, who may have difficulties with language and communication. Therefore, reporting symptoms such as pain can be difficult. In addition, the heterogeneity of our sample population in terms of gastrointestinal symptoms makes assessing improvement difficult. Recommendations for future studies include substantially increasing sample size while simultaneously recruiting set numbers of participants with certain GI symptoms to ensure that significant differences can be seen for each symptom. While the small sample size of our study makes is difficult to reach statistical significance for many of our outcomes, the results confirm that the supplement is well tolerated and justifies larger, better-designed studies examining the effectiveness of the supplement in reducing clinical symptoms of gastrointestinal dysfunction. Sensory studies addressing supplement palatability are necessary for future studies, as many parents reported that their children were sensitive to the taste of the supplement.

## Conclusions

Children with ASD and gastrointestinal symptoms tend to experience gut immune dysfunction and bacterial dysbiosis. Bovine colostrum product appears to be well-tolerated in these children as its own treatment as well as when combined with the probiotic *B*. *infantis*. It is important to note that some of these children experienced improvement in chronic GI symptoms that were not amenable to a number of other common intervention strategies. However, conclusions from this study are limited due to the small sample size and high heterogeneity of symptoms between participants. The limited number of mild side effects coupled with reduced frequency of some GI symptoms in children supplemented with BCP with and without *B*. *infantis* support the need for larger well-controlled trials to determine efficacy of these treatments.

## Supporting information

S1 Fig Proportion of hard and soft stools with treatment. Mean ± SD proportion of total recorded stools that were A) hard consistency (1 or 2 on Bristol Stool Scale) or B) soft consistency (6 or 7 on Bristol Stool Scale) based on stool log data (n = 8 for each group). Significant differences in means (p<0.05) are denoted by an asterisk. D123, days 1, 2 and 3 of the study period (baseline); D835, days 8 through 35 of the study period.
(TIF)

S1 Table Wilcoxon P-values gastrointestinal and behavioral questionnaire data. (DOCX)

S2 Table Non-parametric statistics for stool consistency from stool log data. (DOCX)

S1 CONSORT checklist (DOC)

S1 Protocol (PDF)

S1 Data (XLSX)
