# Supplementary figures and images for: Pilot study of probiotic/colostrum supplementation on gut function in children with autism and gastrointestinal symptoms
Source: PLoS One. 2019 Jan 9;14(1):e0210064. doi: 10.1371/journal.pone.0210064 (PMC6326569; doi:10.1371/journal.pone.0210064)

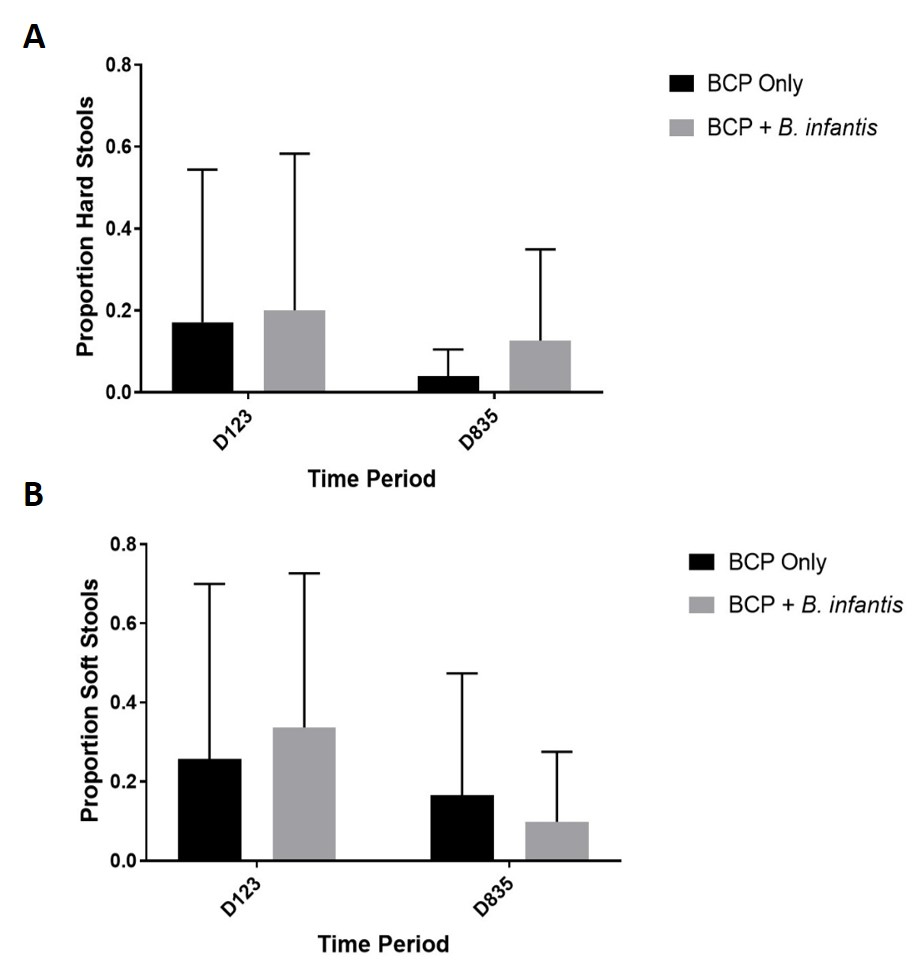

Supplement: S1 Fig — Mean ± SD proportion of total recorded stools that were A) hard consistency (1 or 2 on Bristol Stool Scale) or B) soft consistency (6 or 7 on Bristol Stool Scale) based on stool log data (n = 8 for each group). Significant differences in means (p<0.05) are denoted by an asterisk. D123, days 1, 2 and 3 of the study period (baseline); D835, days 8 through 35 of the study period. (TIF) [file pone.0210064.s001.tif]
