# Supplementary material for: Pilot study of probiotic/colostrum supplementation on gut function in children with autism and gastrointestinal symptoms
Source: PLoS One. 2019 Jan 9;14(1):e0210064. doi: 10.1371/journal.pone.0210064 (PMC6326569; doi:10.1371/journal.pone.0210064)
Supplement: S1 Table — (DOCX) [file pone.0210064.s002.docx]

| **Outcome Variable** | **BCP Only Treatment**  **(n=8)** | **Combination Treatment**  **(n=8)** | **Treatment Comparison**  **(n=8)** |
| --- | --- | --- | --- |
| **Diarrhea**  **Frequency** | **p=0.053** | p=0.203 | P=0.560 |
| **Constipation**  **Frequency** | P=0.423 | P=0.174 | P=0.784 |
| **Pain**  **Frequency** | **p=0.058** | **p=0.048** | P=0.666 |
| **Gas**  **Frequency** | p=0.086 | p=0.120 | P=0.854 |
| **Frequency**  **Normalization** | p=0.789 | p=0.161 | P=0.242 |
| **Consistency**  **Normalization** | **p=0.058** | **P=0.048** | P=0.263 |
| **Weight**  **Change** | P=0.233 | P=0.674 | P=1 |
| **BMI**  **Change** | P=0.439 | P=0.554 | P=0.234 |
| **ABC - Irritability**  **Score Change** | **P=0.021** | P=0.447 | P=0.400 |
| **ABC - Lethargy**  **Score Change** | **P=0.029** | P=0.057 | P=0.528 |
| **ABC - Stereotypy**  **Score Change** | **P=0.013** | P=0.198 | **P=0.037** |
| **ABC - Hyperactivity**  **Score Change** | **P=0.024** | P=0.205 | P=0.123 |
| **ABC - Total**  **Score Change** | **P=0.016** | P=0.310 | P=0.148 |
