# Supplementary material for: Pilot study of probiotic/colostrum supplementation on gut function in children with autism and gastrointestinal symptoms
Source: PLoS One. 2019 Jan 9;14(1):e0210064. doi: 10.1371/journal.pone.0210064 (PMC6326569; doi:10.1371/journal.pone.0210064)
Supplement: S2 Table — (DOCX) [file pone.0210064.s003.docx]

| **Time Periods** | **Proportion Normal Consistency (n=8)** | | | **Proportion Hard Consistency (n=8)** | | | **Proportion Soft Consistency (n=8)** | | |
| --- | --- | --- | --- | --- | --- | --- | --- | --- | --- |
|  | **BCP Only**  **Treatment** | **Combination**  **Treatment** | **Treatment**  **Comparison** | **BCP Only**  **Treatment** | **Combination**  **Treatment** | **Treatment**  **Comparison** | **BCP Only**  **Treatment** | **Combination**  **Treatment** | **Treatment**  **Comparison** |
| **D123 vs D835**  **(Mean Diff**  **[95% CI])** | P=0.402 | **P=0.016** | P=1 | P=0.371 | P=0.855 | P=0.524 | P=0.560 | P=0.281 | P=0.155 |
| **W1 vs D835**  **(Mean Diff**  **[95% CI])** | P=0.272 | P=0.093 | P=0.562 | P=0.100 | P=0.855 | P=0.161 | P=0.834 | P=0.059 | P=0.122 |
| **W1 vs W5**  **(Mean Diff**  **[95% CI])** | P=0.272 | P=0.675 | P=0.451 | P=0.100 | P=0.423 | P=0.188 | P=0.834 | P=0.059 | P=0.350 |
| **D123 vs W5**  **(Mean Diff**  **[95% CI])** | P=0.293 | P=0.178 | P=0.898 | P=0.371 | P=0.423 | P=0.766 | P=0.855 | P=0.201 | P=0.258 |
