# Supplementary material for: Pilot study of probiotic/colostrum supplementation on gut function in children with autism and gastrointestinal symptoms
Source: PLoS One. 2019 Jan 9;14(1):e0210064. doi: 10.1371/journal.pone.0210064 (PMC6326569; doi:10.1371/journal.pone.0210064)
Supplement: S1 Protocol — (PDF) [file pone.0210064.s005.pdf]

A pilot study examining the microbiotal composition in children with autism after use of *Bifidobacterium infantis* and milk oligosaccharides.

1 Protocol Title

A pilot study examining the microbiotal composition of stool in children with autism after use of *Bifidobacterium infantis* and milk oligosaccharides.

2 Author of Protocol

Kathleen Angkustsiri, MD
Paul Ashwood, PhD
David Mills, PhD

3 IRB Review History

This protocol was initially approved on September 16, 2013. It has been assigned IRB#450072.

4 Objectives

AIM 1. To characterize stool microbiotal composition and immune markers after administration of a probiotic with and without a prebiotic in children with autism.

Hypothesis 1.1: Synbiotic administration of *Bifidobacterium infantis* (probiotic) and bovine milk oligosaccharides (prebiotic) will alter the composition of stool microbiota, with greater increases in commensal actinobacteria (including *B. infantis*) and decreases in pathogenic proteobacteria than *B. infantis* alone.

Hypothesis 1.2: Synbiotic administration of *Bifidobacterium infantis* and bovine milk oligosaccharides will alter serum immune (cytokine, immunoglobulin) measures.

AIM 2 (exploratory). To determine tolerability of *Bifidobacterium infantis* and bovine milk oligosaccharides in children with autism.

Hypothesis 2.1: *Bifidobacterium infantis* and bovine milk oligosaccharides will be well tolerated in children with autism, as measured by gastrointestinal and behavioral symptoms.

5 Background

Gastrointestinal (GI) symptoms (most commonly, constipation and diarrhea) are frequently reported in autism (AU)(Buie, Campbell et al. 2010), and biological differences in this subgroup, specifically the bacterial microbiome of the gut (Finegold, Molitoris et al. 2002), may suggest links between the brain and gut via the immune system. Abnormalities in immune function and cytokines have been reported in some children with autism (Ashwood, Anthony et al. 2004) and this may both lead to GI symptoms and be influenced by substances presenting to the immune system from the gut. Immune markers, such as cytokines, can affect the central nervous system through glial cells and alterations in neurotransmitters (Merali, Lacosta et al. 1997). Probiotics (beneficial living organisms) are widely used dietary supplements that are one of the most frequently encouraged complementary alternative treatments by general pediatricians and family practice providers

(Golnik and Ireland 2009). Probiotics can stimulate immunity and modulate intestinal microflora. *Bifidobacterium infantis* is highly present in the intestines of healthy breast-fed infants and decreased in the digestive tract of some children with autism (Finegold, Dowd et al. 2010). It has been beneficial in gastrointestinal disorders such as irritable bowel syndrome and necrotizing enterocolitis (Alfaleh and Bassler 2008). Oligosaccharides are sugars that serve as "prebiotics," stimulating the activity of *B. infantis*. **Our primary goal is to study stool microbiotal composition after the administration of a synbiotic, which is a probiotic (*Bifidobacterium infantis)* in combination with a prebiotic (BMO: bovine milk oligosaccharides).** We will compare microbial composition from baseline in order to determine the effect of the synbiotic. In this pilot study, we also explore if the synbiotic results in greater changes compared with the administration of the prebiotic alone (BMO obtained from bovine colostrum). Serum immune markers will be collected to reflect changes in the intestinal microbiome. Tolerability will be assessed through behavioral measures, including GI symptoms, clinician assessment, and parent questionnaires to provide further support for microbiotal changes.

Probiotics are non-pathological organisms with potentially beneficial effects via immune modulation (Gill and Prasad 2008, Paineau, Carcano et al. 2008) and/or competitive inhibition of other species. Common probiotics include bacteria such as lactobacilli, lactococci, and bifidobacteria and they are frequently present in yogurt and fermented foods (i.e. sauerkraut). Lactobacilli and bifidobacteria are normally present in the GI system soon after birth (Borriello, Hammes et al. 2003). They are considered to have little or no pathologic potential (Mäkeläinen, Tahvonen et al. 2003) and are "Generally Recognized as Safe (GRAS)" (GRAS; http://goo.gl/ljzVO) by the U.S. Food and Drug Administration and have a "Qualified Presumption of Safety (QPS)." Side effects are uncommon and usually mild, including constipation, increased thirst, bloating and gas (McFarland 2006). A workgroup of experts in clinical practice, microbiology, intestinal microecology, pathogenicity, nutrition, toxicology, and public health reported that probiotics are suitable and well tolerated for infants and children (Borriello, Hammes et al. 2003).

There are documented abnormalities in the composition of the microbiota, immune function, and intestinal permeability in individuals with autism. Abnormal microbiota composition in autism includes greater representation of toxin-producing Clostridium species (Finegold, Molitoris et al. 2002) and decreased Bifidobacterium species (Finegold, Dowd et al. 2010, Adams, Audhya et al. 2011). Alteration of the intestinal microbiotal imbalance can alter gut motility and secretion (Critchfield, van Hemert et al. 2011). In addition, reports of elevated levels of antibodies against dietary peptides (de Magistris et al. 2013) and gut peptidases (Vojdani et al. 2004) as well as low digestive enzyme activity (Horvath et al. 1999) and elevated levels of urinary peptides of dietary origin (Reichelt et al. 2003, Reichelt et al. 2009) in some children with autism suggest an impaired capacity for protein digestion. Therefore, exploratory analysis of markers of intestinal permeability (zonulin) (Fasano 2011), pancreatic insufficiency (elastase-1) (Naruse et al. 2006) and microbial putrefaction (protein fermentation metabolites such as ammonia, phenols and sulfides) as well as protein digestion products (peptides) will be assessed in the feces, urine and plasma.

Immune dysfunction and atypical pro-inflammatory cytokine profiles have been documented in autism (Ashwood, Anthony et al. 2003, Ashwood, Anthony et al. 2004, Ashwood and Wakefield 2006) and these are potential mediators between the GI and nervous systems. Cytokines can modulate intestinal epithelial barrier function (“leaky gut”), which is seen in some individuals with autism (D'Eufemia, Celli et al. 1996, Horvath and Perman 2002) and can affect neural signaling through glial cells and/or neurotransmitter metabolism(Merali, Lacosta et al. 1997). Increased gut permeability can also allow for the presentation of antigenic material to trigger an immune response. Both *B. infantis* and Bovine Milk Oligosaccharides (BMO) have been shown to be safe and well tolerated in adults and premature infants without complications (Parente, Cucino et al. 2003, Eiwegger, Stahl et al. 2004, LoCascio, Ninonuevo et al. 2007, Sela, Chapman et al. 2008, German, Freeman et al. 2009, Marcobal, Barboza et al. 2010, Sela, Li et al. 2011, Zivkovic, German et al. 2011). A UCDMC study in premature infants demonstrated that probiotics with fructo-oligosaccharide (FOS) were well tolerated without side effects noted. Currently, the UCDMC NICU is conducting a trial of bifidobacteria and oligosaccharides in premature infants and there is an ongoing study of milk oligosaccharides in healthy adults at the UC Davis Foods for Health Institute.

For description of and previous experience with the product: please see Appendix 1.

6 Inclusion and Exclusion Criteria

Inclusion criteria:

Participants ages 2-11 years of age with a confirmed diagnosis of autism/autism spectrum disorder (ASD) based on the ADI-R and/or ADOS will be eligible. The diagnosis of autism/ASD from another source deemed reliable may also be used. Participants must report the presence of diarrhea and/or constipation based on screening questions from the CHARGE GI questionnaire and a subset of questions from the Questionnaire on Pediatric Gastrointestinal Symptoms-Rome III Version (QPGS-RIII). A reliable caregiver must be available to accompany the child to all visits and oversee proper administration of the synbiotic. Families must agree to not change any interventions during the course of the trial.

Participants meeting all of these criteria may be enrolled provided that they do not meet any of the exclusionary criteria below.

Exclusion criteria:

Participants will be excluded if they have a reliable history of milk protein or other documented food allergy. Exclusionary medical conditions include, but are not limited to: compromised immunity, GI conditions (inflammatory bowel disease, celiac disease, short gut, etc.), systemic steroid, antifungal, or antibiotic use within the past month, failure to thrive, and individuals on medically prescribed diets or supplements (including probiotic use within the past month) other than a standard multivitamin. Children with other medical conditions, such as uncontrolled seizure disorder, genetic disorders (fragile X syndrome, tuberous sclerosis, etc), liver/pancreatic disease, cystic fibrosis, or other serious medical condition determined by the study

physician will also be excluded. Medications that may interfere with intestinal mobility or microbiota composition will also not be allowed. Participants with a full scale IQ <40 will be excluded. A prior IQ score from a source deemed reliable may be used. (For example, from a licensed physician, psychologist, psychometrist or other qualified professional.)

Participants will be pre-screened for eligibility in this study by phone using the study's oral consent form. Oral consent forms will be reviewed by Dr. Angkustsiri before for final approval for enrollment in the study.

7 Study-Wide Number of Subjects

We intend to enroll twenty subjects in hopes of having ten subjects complete the protocol.

8 Study-Wide Recruitment Methods

Twenty children with autism/ASD and irregular bowel movements ages 2-11 years of age will be recruited for this study. Recruitment strategies will include solicitation of families who have participated in Dr. Ashwood's Autism Speaks study "Defining the Underlying Biology of Gastrointestinal Dysfunction in Autism," all of whom participated in the CHARGE (Childhood Autism Risks from Genetics and the Environment) study and received autism diagnostic assessments. Additionally, only families that indicated a desire to be contacted for future studies will be solicited.

We will also contact families who have participated in prior research at the MIND Institute and who have agreed to be contacted for future studies through the MIND Research Volunteer Registry. These families will initially be contacted either by phone or email and asked to participate in an oral consent questionnaire to evaluate eligibility. A flyer explaining the study will also be available to families in clinic. Upon completion of the questionnaire, and approval from Dr. Angkustsiri, the participant and his or her family will be invited to complete a screening visit for the study.

Upon successful completion of the screening visit, participants will be asked to begin the trial.

9 Study Timelines

Screening and enrollment will begin upon IRB approval of this protocol and associated documents. Enrollment will continue for approximately two months in order to reach the full enrollment.

Each participant will be involved in this trial for a period of twelve weeks. This will include four visits to the UC Davis MIND Institute and two phone call checks. See Table 1 listed on page 7 for

procedures Involved.

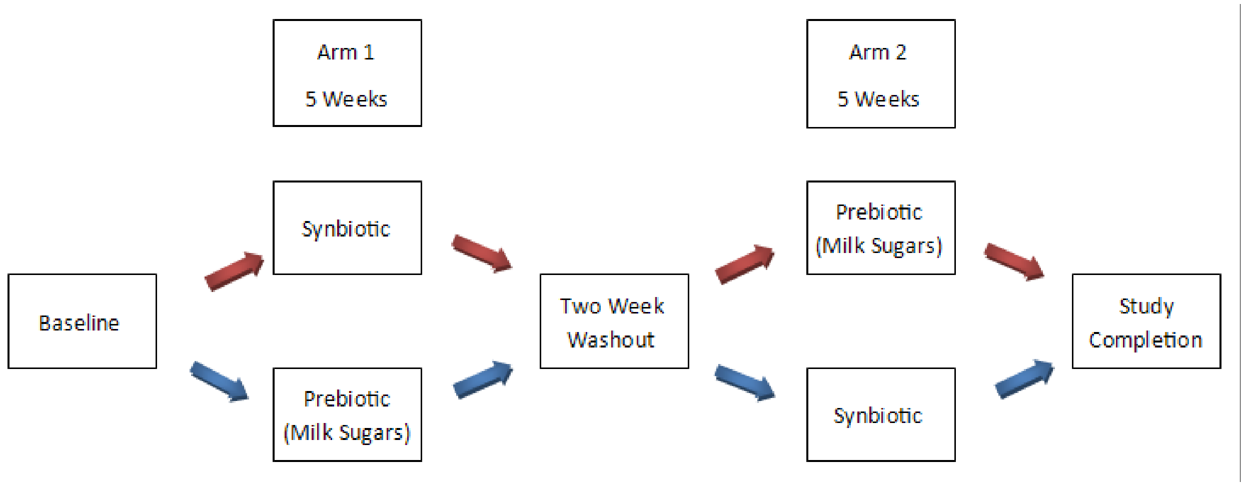

Figure 1: Study diagram

Participants will be active in this trial until about February, at which point the final participant will have completed the trial period.

Data analysis of data collected during the trial period of each participant will be analyzed after the final participant has completed the trial, which is expected to be about March 2014.

10 Study Endpoints

The primary endpoint will be change in intestinal microbiotal composition. Secondary endpoints will include change in serum immune markers, fecal and plasma zonulin, fecal elastase-1, fecal and urinary protein/peptide profiles, and fecal and urinary microbial putrefaction metabolites.

Tolerability will be monitored through GI questionnaires, behavioral questionnaires, and clinician assessment.

11 Procedures Involved

This pilot study is a randomized, controlled trial of a synbiotic (BMO + *B. infantis)* vs. BMO alone.

Synbiotic/BMO only administration

Each participant will be randomized by the IDS pharmacy to receive either the synbiotic first or the BMO first in a cross-over study design. This is a double blind study in that both participants and study personnel will not be aware of which treatment arm the participant will receive first. The study supplement will be dispensed in identically packaged pouches. The study will last 12 weeks, including 5 weeks of BMO use, 5 weeks of the synbiotic, and a two-week washout period in between.

The synbiotic and the BMO will be given once a day. Each synbiotic dose will be **100 mg of *B. infantis, or* 10 Billion CFU ($1x10^{10}$), delivered once a day with 0.15 g/pound body weight per day of BMO.** The **BMO only arm** will be the identical dose of 0.15 g/pound body weight/day BMO given orally. The synbiotic or BMO will be a powder the parent can deliver in many different recipes including any cold milk-based liquid, ice-cream or pudding. Parents will be provided an individualized recipe booklet that provides dosing strategies and instructions as well as a magic bullet blender to facilitate supplement incorporation into food items. Parents will also be instructed to store the study drug in the freezer and to ensure the child consumes the entire supplement dose within 20 minutes.

Stool Collection

Baseline stool samples will be collected within 48 hours prior to the first dose of synbiotic. Subsequent samples will be taken after completing the first study arm at week 5, after completing the washout period at week 7, and after completing the second arm of the study at week 12.

Families will be given a kit including a styrofoam container with an ice pack, and a sterile tongue blade. Stool samples will be collected at home by the parent prior to their study visit into a sterile specimen container using a sterile tongue blade and kept cold in either a styrofoam transport container with ice pack or their home freezer until the visit.

Stool samples will be analyzed for microbiotal composition, microbial metabolites, peptide profile, intestinal permeability (zonulin), and pancreatic insufficiency (elastase-1).

Urine Collection

Urine will be collected at each visit for exploratory analysis to characterize microbial metabolites as a biomarker of intestinal microbiota as well as peptide profiling. Parents or caregivers will be asked to assist in collecting a first morning void. Children who are not developmentally able to void at will shall be given the option to use a bag for collection. Upon collection, parents or caregivers will be asked to store the sample in either their home freezer or in the provided styrofoam transport container with ice pack. Samples will be stored long term at -80°C until analyzed by mass spectroscopy and NMR for metabolomics and peptidomics.

Blood Draw

Blood will be drawn from patients at each visit. Blood will be drawn on site at the UC Davis MIND Institute by a study physician or certified phlebotomist experienced in working with children with neurodevelopmental disorders. Up to 28 mL of blood will be collected at each draw. . Cytokines will be measured by Luminex technology in the Ashwood laboratory to determine profiles of pro-inflammatory (IFNg, IL-1, IL-6, IL-13 and TNFa) cytokines and anti-inflammatory (IL-10 and TGFb1) cytokines. Following the synbiotic, we expect to see increases in anti-inflammatory cytokines. We will also determine immunoglobulin levels (IgA, IgG and IgM) by ELISA. In previous

studies we have seen that immunoglobulin levels are decreased in children with autism; following synbiotic administration, we expect to see levels increase to those seen in typical developing children of the same age. The blood draw will also include a CBC (Complete Blood Count) for safety as well as plasma biomarkers of microbial metabolites and intestinal permeability (zonulin).

Table 1. Study visits, assessments, and procedures.

	Week 0	Week 2/3	Week 5	Week 7	Week 9/10	Week 12
Assessment	Baseline Visit	Call 2	Visit 3	Visit 4	Call 2	Visit 4
ADOS/ADI-R	Obtained Previously From A Reliable Rater					
IQ / Devlopmental Testing	Obtained Previously From A Reliable Rater					
ABAS-II	X					
Physical Exam/ Vitals	X		X	X		X
Adverse Events	X	X	X	X	X	X
GI Questionnaires	X		X	X		X
ABC	X		X	X		X
RBS-R	X		X	X		X
Stool Sample	X		X	X		X
Urine Sample	X		X	X		X
Blood Draw	X		X	X		X

Adaptive Behavior Assessment System – Second Edition (ABAS-2)

The ABAS-2 is composed of three domains, including conceptual, social, and practical. The domains are sub-divided into ten different skills areas that generate a norm-referenced standard score. The ABAS-II has been previously approved by the IRB as part of the MIND Institute Protocol Repository (MIPR, IRB#200513093).

Medical History and Physical Exam

During visits to the UC Davis MIND Institute, participants will undergo a physical exam and answer questions about their medical history. These appointments will include a review of adverse events for returning participants. Adverse events will also be queried via phone call throughout the study.

GI questionnaires

CHARGE Gastrointestinal History Questionnaire

The GI History includes 10 Likert scale items (0=never; 1=rarely; 2=sometimes; 3=frequently; 4=always) for each current and past (last 3 months) gastrointestinal symptom (abdominal pain, gaseousness/bloating, diarrhea, constipation, pain on stooling, vomiting, sensitivity to foods, difficulty swallowing, blood in stool and blood in vomit). The CHARGE GI History Questionnaire is included in this submission.

Questionnaire on Pediatric Gastrointestinal Symptoms (QPGS-RIII)

The QPGS-Rome III, is a parent-report measure of gastrointestinal symptoms scored on a 5-point scale and assesses functional gastrointestinal disorders based on the Rome III criteria. Only a specific subset of the questions on the QPGS-RIII will be used as part of this study, as conditions of interest for this study include functional diarrhea, functional constipation, and irritable bowel syndrome. This subset of questions can be found in the Oral Consent form, and the same questions will be used throughout the study as a measure of gastrointestinal function.

Aberrant Behavior Checklist (ABC)

The ABC is a parental survey consisting of 58 questions designed to measure the severity of autism-associated behaviors, including irritability, lethargy, stereotypy, hyperactivity and inappropriate speech. The irritability subscale includes self-injurious and aggressive behavior. The total score range within the ABC is 0–174, with higher scores indicating more severely affected behavior. The ABC has been previously approved by the IRB as it is part of the MIND Institute Protocol Repository (MIPR, IRB # 200513093).

Repetitive Behavior Scale-Revised (RBS-R)

The RBS-R is a 44-item self-report questionnaire that is used to measure the breadth of repetitive behavior in children, adolescents, and adults with Autism Spectrum disorders. The RBS-R consists of six subscales including: Stereotyped Behavior, Self-injurious Behavior, Compulsive Behavior, Routine Behavior, Sameness Behavior, and Restricted Behavior. Behaviors are rated on a 4-point scale: 0-Behavior does not occur, 1-Behavior occurs and is a mild problem, 2-Behavior occurs and is a moderate problem, 3-Behavior occurs and is a severe problem. The RBS-R has been previously approved by the IRB as it is part of the MIND Institute Protocol Repository (MIPR, IRB # 200513093).

Bristol Stool Form Scale

The Bristol Stool Form Scale is a seven point visual scale used to classify stool consistency. Parents will be asked to keep track of their child’s bowel movements throughout their participation

in this study (as asked on a stool log), and to record each stools' consistency as described by this scale.

12 Data and Specimen Banking

Each sample will be coded by a unique numerical identifier.

Biological samples (blood, urine and stool) will be kept for a maximum of ten years and will be destroyed in accordance with protocols in place for biological samples. Stool will be autoclaved prior to disposal. Urine will be neutralized with bleach to make a 10% bleach solution that will incubate at room temp for 30 minutes prior to pouring down the sink with cold running water.

Blood: Blood will be drawn at four points throughout the study with a total blood draw volume of up to 28 mL at each draw. Serum samples will be delivered daily to Dr. Ashwood's lab located in the Wet Lab at the MIND Institute.

Urine: Urine samples will be collected at four points throughout the study. Samples will be stored at -80°C until analyzed.

Stool: Patient's caregivers will be asked to collect a stool sample just prior to each visit requiring such a sample. Caregivers will be given a kit (a small styrofoam container with a cold pack, sterile tongue blade, and a sterile specimen container). Caregivers will be asked to keep the cold pack in their home freezer until a sample is obtained (generally no more than 36 hours prior to a scheduled visit, 48 hours at the most). The sample will be stored in the styrofoam container with the cold pack until it is brought in during their next visit. Upon obtaining the stool sample from the caregivers, the samples will be frozen at -20°C and transported to Dr. Mills' lab weekly where samples will be kept frozen at -80°C.

Stool samples will be picked up weekly by a member of Dr. Mills' lab, or study personnel from the MIND will deliver the specimens to Dr. Mills' lab on ice. In the Mills lab, stool will be extracted and 16S rDNA-based PCR primers for total bacteria and total bifidobacteria will be used to determine the percentage of bifidobacteria present. Species specific bifidobacteria primers will then be used to determine whether the administered strain is the dominant stool strain (groups 3-8) and whether the administered oligosaccharide stimulates a specific strain (groups 1, 2, and 5-8).

13 Data Management

We will use the REDCap (Research Electronic Data Capture) system for data management, which provides secure, web-based applications that are flexible enough to be used for a variety of types of research, provide an intuitive interface for users to enter data and have real time validation rules (with automated data type and range checks) at the time of entry. These systems offer easy data manipulation with audit trails for reporting, monitoring and querying patient records, and an automated export mechanism to common statistical packages (SPSS, SAS, Stata, R/S-Plus). REDCap

servers are housed in a local data center at UC Davis Health System and all web-based information transmission is encrypted. REDCap was developed specifically around HIPAA-Security guidelines. REDCap has been disseminated for use locally at other institutions and currently supports 240+ academic/non-profit consortium partners on six continents and over 26,000 research end-users (www.project-redcap.org).

The subjects' health information, along with the identifiers, will be kept with the investigator until the conclusion of the study, or when immediate access is no longer required. Thereafter, the information may be transferred to a records and information management company for long-term storage, or to a UCDMC long term storage facility. When the investigator no longer require the information (but no sooner) than 5 years, the documents will be securely shredded. All study personnel will have access to study records, data, and specimens. Every attempt will be made to ensure that the personal and medical information of the subject will be kept private.

14 Provisions to Monitor the Data to Ensure the Safety of Subjects

This study is low risk. The products used in this study are dietary supplements designed to balance the intestinal flora and are not intended to "diagnose, cure, mitigate, treat or prevent disease." Any evidence of illness or infection will be thoroughly evaluated.

Dr. Angkustsiri, a board-certified developmental-behavioral pediatrician, will monitor all reported adverse events and Dr. Randi Hagerman and Dr. Lauren Plumer, two pediatricians not involved in the study, will be available to informally review adverse events.

Adverse events will be queried as part of the medical history at baseline and weeks 5, 7, and 12, as well as through phone calls on weeks 2/3 and 9/10. Any adverse events that could be related to the study will be reported to the IRB as required by IRB guidelines.

15 Withdrawal of Subjects

The study team reserves the right to withdraw subjects from the study for reasons including, but not limited to, adverse events and non-compliance. The caregivers of a subject may also withdraw their son or daughter from the study.

If a subject is withdrawn from the study early, caregivers will be asked to return to the MIND Institute for a termination visit which will include a visit with a study physician who can assess for adverse events and general safety of the subject.

Information gathered about a subject who has terminated the study early, as well as any blood and stool samples, will be kept for analysis unless the subject's caregivers specifically ask for this information to be removed for analysis. Caregivers will be informed of this in the event of an early termination.

16 Risks to Subjects

The most common anticipated risks due to participation in the study include anxiety, frustration, fatigue, or embarrassment during the answering of questionnaires, study assessments

and testing, as well as during the medical history and exam. Breaks will be offered to subjects as needed.

Bifidobacteria are abundant in the large intestines soon after birth (Borriello, Hammes et al. 2003). Probiotics are considered to have little or no pathologic potential (Mäkeläinen, Tahvonen et al. 2003) and have been used safely in children with few side effects (Zoppi, Cinquetti et al. 2001)(Gopal, Prasad et al. 2003) at doses similar to those in the proposed study. Previous studies have also documented the tolerability and safety of BMO (Buddington, Williams et al. 1996, Kleessen, Sykura et al. 1997, Pedersen, Sandström et al. 1997, Bouhnik, Vahedi et al. 1999, Kruse, Kleessen et al. 1999, Bouhnik, Raskine et al. 2004, Puccio, Cajozzo et al. 2007, Ziegler, Vanderhoof et al. 2007, Chouraqui, Grathwohl et al. 2008, Silk, Davis et al. 2008). The potential side effects associated with synbiotics may include: vomiting, belching, bloating, flatulence, diarrhea, rumbling stomach, and stomach cramps. Infection is an extremely rare and unlikely side effect (Gasser 1994, Saxelin, Chuang et al. 1996), with estimates that they may represent up to 0.05%–0.4% of cases of infective endocarditis and bacteremia (Borriello, Hammes et al. 2003). Existing data suggest that the risk of bacteremia is <1 case per million individuals (Borriello, Hammes et al. 2003). Any immunocompromised individuals will be deemed ineligible, although probiotics have been considered safe to use even in individuals with HIV (Wolf, Wheeler et al. 1998, Cunningham-Rundles, Ahrné et al. 2000). Phone calls will be conducted throughout the study to assess adverse events. Dr. Angkustsiri's contact information will be provided to all subjects' caregivers for emergencies related to the study.

A potential risk is the chance of infection or bleeding at the site where the venous blood draw is completed. This site will be carefully cleaned prior to the draw and an appropriate dressing will be applied to the area.

Every attempt will be made to ensure that the personal and medical information of the subject will be kept private; however, we cannot guarantee total privacy. The subject's personal and medical information may be given out if required by law. All the data obtained from this study will be kept confidential. Only research personnel on the IRB approved protocol will have access to the data. Electronic data will be password protected and physical data will be stored in a locked file room.

Because this study involves young children between the ages of 2 and 11 years old, all personnel associated with the study will pay special attention to all potential risks associated with the study.

Psychological, social, or legal injury is not anticipated.

17 Potential Benefits to Subjects

This study aims to characterize the microbiotal composition of stool in children with autism or ASD after synbiotic administration without the specific goal of clinical improvement. As such, each subject may not personally receive any direct benefit from participation in this study. Participants have been provided with this information in the consent form.

Despite not offering direct benefits to patients, participants will undergo neuropsychological assessments, the results of which may be made available to the family of participants on request. Participants will also benefit from the medical exams offered through the study. Additionally, a complete blood count will be conducted as a part of this study. Participants will be informed of clinically significant findings from either the medical exam or CBC as appropriate.

18 Vulnerable Populations

This study intends to enroll developmentally disabled children with autism or ASD who will be too young to consent to participate to this study. As such, reliable and competent caregiver(s) (such as (a) parent(s) or other legal guardian) must give consent to participate in this study. The caregivers of the participant will be informed of all known risks and potential benefits associated with the study.

Because this study involves young children between the ages of two and eleven years old with developmental disorders all study staff will pay special attention to the potential risks associated with the study. We have done research with developmentally disabled and cognitively impaired individuals of all ages for many years. Consent for individuals with cognitive disability is always signed by parent or caregiver, and no study-specific procedures will be done for any subject prior to consent.

A letter of information will be supplied and explained to all participants who are between the ages of 8 and 11 years old (inclusive). As this study is not open to subjects aged 12 and older, the study does not have an Assent Form available.

19 Multi-Site Research

This is a single site research study.

20 Community-Based Participatory Research

Not applicable.

21 Sharing of Results with Subjects

The data gathered regarding microbiotal composition will be for research purposes and will be of limited use to the families. Thus, data collected throughout this study will not be made available with the exception of developmental/cognitive and/or adaptive scores if requested. These scores will be released to the families in the form of a research report.

Study participants will be unblinded as to which treatment was received during each arm of the trial when the last participant has completed the trial. Unblinding will be conducted by a study physician.

22 Setting

The UC Davis MIND Institute

We will recruit and patients from existing MIND Institute research studies and the Volunteer Research Registry. Participants will be evaluated at the MIND Institute Research Clinic. The MIND Institute is a multi-purpose research facility focusing on patients with autism spectrum disorders and other neurodevelopmental disorders. The MIND Institute is well equipped with multiple patient exam rooms available for questionnaires, surveys, and physical exams. This will be the main study site, as recruitment, screening, and patient visits will occur at the MIND Institute. The use of space at this facility has been pre-approved by the MIND MICRO committee.

In order to ensure that multiple visits to the MIND Institute is not a barrier for participating in the study, home visits for some assessments will be offered to families who would otherwise not be able to participate in the study.

Dr. David Mills Lab

Dr. Mills has a well-equipped anaerobic microbiology lab and has previously evaluated a wide variety of bifidobacteria species including the ability of specific micro-organisms to grow in oligosaccharide medium. Dr. Mills' lab also has thermocycler equipment to quantify bifidobacteria. The Mills laboratory is in the new Robert Mondavi Institute for Wine and Food Science at UC Davis. This includes a ~1000 square-foot lab equipped for all proposed microbiological and biochemical work. The laboratory is equipped with a specially designed Coy anaerobic chamber with incubator, a BioTek PowerWave 340 plate reader and all the necessary tools to perform the prebiotic screening and microbial BMO-functionality assays. Existing equipment that will be used in this project are: incubators, 2 anaerobic hoods, shakers, biosafety hood, ovens, UV-Vis spectrophotometers, electrophoresis apparatus, gel documentation equipment, refrigerators, freezers, cell disrupters, microscopes, table top centrifuge, microcentrifuges, high-speed centrifuge, several -80C freezers, 2 real time thermocyclers, and 2 microtiter plate readers for bacterial growth studies. The laboratory is equipped with five Macintosh computers, two Pentium PC computers, and several common printers.

Dr. Paul Ashwood's Lab

The laboratory facilities of Dr. Ashwood are housed on the second floor of the MIND Institute Wet laboratory building on the UC Davis Medical Center Campus in Sacramento, CA. The Ashwood laboratory consists of 600 sq feet of wet laboratory space dedicated to immunological analysis and contains instrumentation and facilities for this function. Further resources include an additional 200 sq feet space dedicated to the housing of a BD Biosciences LSRII flow cytometer and

associated computational instrumentation, plus a Luminex workstation and instrumentation for multiplexing technology. Within the research wet laboratory building all important facilities are present including computer and internet hookups, tissue culture room, dark room, and common equipment rooms. The research wet laboratory building is adjacent to the MIND clinical facility. Dr. Ashwood's laboratory has all the major equipment required for immune-based studies, including a BD LSRII flow cytometer equipped with a solid-state Coherent Saphire100mW 488nm laser (blue), a Coherent radius 25mW 640nm HeNe laser (red), and a solid-state 50mW 405nm laser (violet) and 11 PMTS for enhanced fluorochrome color detection. The LSRII flow cytometer instrumentation is supported by a dedicated PC workstation, software and printer. Further instrumentation includes: Luminex100 with a dedicated PC workstation and software, Victor3V ELISA plate reader with a dedicated PC workstation and software, Baker Bioguard laminar flow hoods, high speed centrifuges, pH meters, analytical balances, Zeiss fluorescent microscope, two Hereaus CO2 incubators, refrigerators, -20° and -80°C freezers. Additional office space is dedicated for data analysis and data management. Additional support core facilities that house additional refrigerators, freezers, photographic development facilities, autoclaves and facilities for washing glassware are available within the Wet laboratory facility at the MIND Institute.

23 Resources Available

For a full list of research personnel associated with this study, please refer to the associated personnel list.

The following personnel have prominent roles in this study, which are described below:

Kathleen Angkustsiri, MD

Dr. Angkustsiri will be the Principle Investigator for this study. She will oversee the informed consent process, follow participants through their progression in the study, and be responsible for data analysis. She will also conduct physical exams for each patient. Dr. Angkustsiri has extensive training and experience with autism and genetic disorders that cause developmental delay and has been PI of a CMN grant "Biological Predictors of Functional Outcome in Children with Chromosome 22q11.2 Deletion Syndrome" and co-PI on targeted treatments for fragile X syndrome and autism.

David Mills, PhD

Dr. Mills is the Peter J. Shields Endowed Chair in Dairy Sciences with over twenty years of experience in working with bacteria active in intestinal health. Dr. Mills will oversee analysis of stool microflora and has a well-equipped anaerobic microbiology lab to quantify bifidobacteria (TRFLP

and PCR). He has previously evaluated a wide variety of bifidobacteria species including the ability of specific micro-organisms to grow in oligosaccharide medium.

Paul Ashwood, PhD

Dr. Ashwood will oversee the immunological analysis. He is a leading world expert on the impact of immune dysfunction in autism with academic training is in pharmacology, toxicology/pathology and immunology. His lab is housed at the UC Davis MIND Institute Wet Lab, and is well equipped for this analysis.

The UC Davis Medical Center Investigational Drug Services pharmacy will store and dispense the study drug.

Study physicians include **Drs. Angkustsiri, Leigh, Plumer, , and Lozano**.

Study coordinators include **Jonathan Polussa, Lindsey Partington, Erika Bickel, Megan Sanctuary,** and **Laura Berkowitz-Sutherland**, will be responsible for contacting and screening potential patients, scheduling and coordinating patient appointments and visits, coordinating the processing and storage, meeting with study monitors, and organizing relevant supplies and data for the study. Each of these coordinators has prior experience as a research coordinator for studies involving autistic patients.

Database management will be overseen by a member of the **CTSC Bioinformatics Program**. A request for these resources has already been approved by the CTSC and we are awaiting assignment of this technician.

All study personnel will be familiar with the protocol and research procedures.

The **UC Davis MIND (Medical Investigations of Neurological Disorders) Institute** was launched in 1998 as a unique interdisciplinary institute to study autism and other neurodevelopmental disorders. The basic (wet labs) and clinical buildings, total approximately 100,000 ft^2, were completed in 2002 and are co-localized on the UC Davis Medical Center campus in Sacramento. The MIND Institute is a collaborative endeavor among parents, community leaders, and researchers and clinicians at UC Davis. The Institute takes a three-pronged approach to solving the complex problems posed by disorders of the brain by bringing together resources at UC Davis, within the community, and from the broader UC system to focus on basic and clinical research, patient treatment, and the education of patients, families, and providers.

Computers: All key personnel have up-to-date personal computers with high-speed internet and printer connections.

Offices: The MIND. Institute has office space available for the Center's administrative assistant. All office space includes up-to-date computers, high-speed internet access, printers and telephones with private voice mail.

Evolve Biosystems manufactures proprietary and targeted blends of synbiotic products that contain metabolically active probiotics with matched human equivalent prebiotics based on research from the University of California Davis Foods for Heath Institute.

Evolve Biosystems is using a contract manufacturer to produce the *B.infantis* for this study. The contract manufacturer is Culture Systems, Inc, founded in 1991. Culture Systems Inc. focuses on research and development, marketing, and manufacturing of dairy and probiotic cultures. Culture products from their plant have been used in the food industry since its inception. They produce all products under GMP practices, receive inspections from the health department and FDA, and issue a Certificate of Analysis for each batch including pathogen control as part of our QC process. Their facilities include 4 industrial fermentation/harvesting systems and several freeze-driers in 18,000 SQ of space. They currently manufacture cultures for several dairy companies and animal feed companies. They are capable of producing various lactic cultures.

Sterling Technologies has GMP, dairy and food grade production facilities in South Dakota that has a capacity to process 105,000 gallons of colostrum per week. The colostrum product is a commercial product produced under GMP conditions. The whole colostrum is defatted and the casein removed to produce the product being used here. Separation of casein and whey fractions is standard practice in the dairy industry. Certificate of analysis is provided for each batch of product. Stimulac has been sold into the human nutrition market as a dietary supplement for 25 years. The bovine colostrum product has been has been sold as an dietary supplement into the autistic community for the past 15 years in aiding the immune system and enteric system of children with autism sold under the trade name ColostrumGold. This is a liquid product bottled in 4 oz containers that must be refrigerated after opening. Sterling has agreed to freeze dry the prebiotic product for this study.

24 Prior Approvals

The MIND Institute Pilot Grant Awards Committee approved funding for this study on November 27, 2012.

25 Recruitment Methods

Twenty children with autism/ASD and irregular bowel movements ages 2-11 years of age will be recruited for this study in hopes of having ten children complete the protocol. Recruitment strategies will include solicitation of families who have participated in Dr. Ashwood's Autism Speaks study "Defining the Underlying Biology of Gastrointestinal Dysfunction in Autism," all of whom participated in the CHARGE (Childhood Autism Risks from Genetics and the Environment) study and received autism diagnostic assessments. Additionally, only families that indicated a desire to be contacted for future studies will be solicited.

We will also contact families who have participated in prior research at the MIND Institute and who have agreed to be contacted for future studies through the MIND Research Volunteer Registry. These families will initially be contacted either by phone or email and asked to participate in an oral consent questionnaire to evaluate eligibility. A flyer explaining the study will also be made available to families in clinic. Upon completion of the questionnaire, and approval from Dr. Angkustsiri, the participant and his or her family will be invited to complete a screening visit for the study.

Participants will receive a total of $85 for participating in the study. Of this, $50 will be in the form of gift cards, the remaining $35 will come in the form of a magic bullet blender, with which the family is able to mix the study supplement into food or drink for consumption throughout the study. The family is able to keep after the study.

26 Local Number of Subjects

This study intends to have 10 subjects complete the study protocol, and we expect to enroll up to twenty subjects to account for screening failures and early terminations.

Power Analysis: The proposed two-period crossover study design with baseline and follow-up measurements for each period will enable each child to serve as their own control for purposes of quantifying the effects of synbiotic on microbiotic outcomes. The effect size parameter of interest will be the mean within-child between-period (synbiotic vs. prebiotic) difference in within-period change (from baseline to follow-up). With an analysis of ten children, the proposed study design will allow us to estimate the parameter of interest with sufficient precision that the margins of error (i.e. halfwidths of 95% confidence intervals) will be 1.4 root mean square error (rmse) wide. In addition, the study will have **80% power** to detect differences of magnitude 1.94 rmse, under two-sided testing (with alpha=5%) of the null hypothesis that the effect size is 0. The rmse is the square root of the residual error variance component. The residual error variance component is equal to (1 - R) * V, where R is the within-child correlation and V is the total variance of the outcome distribution. To our knowledge, no estimates are available of the within-child correlation for the proposed outcomes in this clinical population. (Indeed, this is one of the knowledge gaps that the pilot study will aim to fill.) However, Dr. Mills lab have measured similar bifidogenic response in infants, for whom the within-infant correlations have ranged as high as 40%. Although it is plausible

to assume that correlations will be larger in older children, we conservatively assume a within-child correlation of 40%. Using the above expressions, we anticipate the margin of error of our parameter estimate to be 1.1 standard deviations (SD) and the minimum detectable difference to be 1.5 standard deviations, where the standard deviation is the square root of the total variance V. In case the correlation is as high as 70%, the margin of error and the minimum detectable difference will be less than 0.7 and 1.1 SD, respectively. For the range of correlations that we considered, from 0.4 to 0.7, the proposed study will yield sufficiently precise estimates to achieve the research objectives of the proposed pilot study and develop evidence that can be used to inform future research in this population.

We believe that we will be able to recruit all of our participants locally given our access to the thousands of families with autism spectrum disorders that have participated in research at the MIND Institute.

27 Confidentiality

Research charts will be kept on the subjects and subject information will be coded to protect confidentiality. All charts will be kept in locked cabinets. The identifiers used to identify subjects will be kept in locked offices and/or locked cabinets, and the electronic database containing personal information will be kept on a secure computer network accessible only to PI's research team. If information from the study is published or presented at scientific meetings, subject names and other information that could identify subjects, will not be used.

Specimens will be transported daily by study personnel to Dr. Ashwood's team at the MIND Institute wet lab and where they will be stored until analysis. A member of Dr. Mill's team will pick up the stool samples weekly and transport them on dry ice to the Mills' lab where they will be stored at -80C.

28 Provisions to Protect the Privacy Interests of Subjects

As this trail will involve young, developmentally delayed children, extra attention will be given when the study requires potentially uncomfortable procedures. Efforts will be made to minimize discomfort.

If possible, subjects and their families will be limited in the number of different types of staff they interact with during the trial. For instance, subjects and their families will work with the same study coordinator throughout the trial period and see the same study personnel throughout the trial.

Subjects and their caregivers will have access to contact information for their coordinator and study physician, and will be encouraged to express any questions or concerns they have.

Special care will be taken when a procedure is known to cause discomfort to subjects and/or their caregivers. In these instances, extra time will be taken to explain the procedure and guide them through the study requirements as needed, and extra time will be taken to explain the procedure and subjects will have each step explained.

In severe cases, the study physician and/or the principle investigator may determine that the discomfort experienced may create a situation where the procedure (or the study as a whole) is no longer in the subject's best interest. Should such a situation arise, alternatives will be discussed with study personnel as well as caregivers and the subject as appropriate an applicable.

29 Compensation for Research-Related Injury

We do not anticipate any research-related injuries through this study. However, should such injury occur as a direct result of participation in this study, the University of California will provide necessary medical treatment. The cost of the treatment may be covered by the University, or may be billed to a participant's insurance company. Subjects will not be compensated above what would be required to treat any injury.

30 Economic Burden to Subjects

Participation in this study does not incur direct a direct economic burden to subjects, as there will not be any costs associated with participation. There may be indirect costs associated with participation including travel and time taken off work to meet appointments.

31 Consent Process

The study consent form will be made available to subjects prior to visiting the MIND Institute

Consenting will take place at the UC Davis MIND Institute or by phone by research personnel approved by the PI to have in-depth knowledge of the protocol and related procedures. Participants will be screened for this study and be informed of their eligibility at least one week prior to consent. Participants will also have access to the study consent form.

For participants who are consented by phone, the consent form will be signed by parents or legally authorized representative. A copy of the form may be faxed to the study team, and the family will be asked to bring the original copy when visiting the MIND Institute.

Procedure for obtaining consent will be followed as outline in HRP-090 Consenting and all study measures and assessments will be done in English, as some of the measures used through this study are only available in English (including the Repetitive Behavior Scale-Revised and the Aberrant Behavior Checklist).

All participants will be under the age of 18, and will require the consent of a parent, guardian, or other legally responsible adult with documentation of authority to sign on behalf of the participant in order to be enrolled in this study.

Consent to enroll in this study will preferentially be obtained from both parents or guardians of the participant, however, only one parent will be required to consent to participate in this study.

32 Process to Document Consent in Writing

Consent will be documented on the attached Consent Form in accordance with procedures outlined in HRP-091.

33 Drugs or Devices

Not applicable.

We have contacted the FDA, and Dr. Cara Fiore from the Center for Biologics Evaluation & Research has determined that this study does not warrant an IND. We have attached documentation of this determination.

The products used in this study are dietary supplements designed to balance the intestinal flora and are not intended to "diagnose, cure, mitigate, treat or prevent disease". Ongoing studies in the UCDMC NICU and UC Davis Food Science and Technology Department have IRB approval for the use of similar products without the need for an IND.

References

Adams, J. B., T. Audhya, S. McDonough-Means, R. A. Rubin, D. Quig, E. Geis, E. Gehn, M. Loresto, J. Mitchell, S. Atwood, S. Barnhouse and W. Lee (2011). "Nutritional and metabolic status of children with autism vs. neurotypical children, and the association with autism severity." Nutr Metab (Lond) **8**(1): 34.

Alfaleh, K. and D. Bassler (2008). "Probiotics for prevention of necrotizing enterocolitis in preterm infants." Cochrane Database Syst Rev **1**.

Ashwood, P., A. Anthony, A. A. Pellicer, F. Torrente, J. A. Walker-Smith and A. J. Wakefield (2003). "Intestinal lymphocyte populations in children with regressive autism: evidence for extensive mucosal immunopathology." J Clin Immunol **23**(6): 504-517.

Ashwood, P., A. Anthony, F. Torrente and A. J. Wakefield (2004). "Spontaneous mucosal lymphocyte cytokine profiles in children with autism and gastrointestinal symptoms: mucosal immune activation and reduced counter regulatory interleukin-10." J Clin Immunol **24**(6): 664-673.

Ashwood, P. and A. J. Wakefield (2006). "Immune activation of peripheral blood and mucosal CD3+ lymphocyte cytokine profiles in children with autism and gastrointestinal symptoms." J Neuroimmunol **173**(1-2): 126-134.

Borriello, S., W. Hammes, W. Holzapfel, P. Marteau, J. Schrezenmeir, M. Vaara and V. Valtonen (2003). "Safety of probiotics that contain lactobacilli or bifidobacteria." Clinical infectious diseases **36**(6): 775-780.

Bouhnik, Y., L. Raskine, G. Simoneau, E. Vicaut, C. Neut, B. Flourié, F. Brouns and F. R. Bornet (2004). "The capacity of nondigestible carbohydrates to stimulate fecal bifidobacteria in healthy humans: a double-blind, randomized, placebo-controlled, parallel-group, dose-response relation study." The American journal of clinical nutrition **80**(6): 1658-1664.

Bouhnik, Y., K. Vahedi, L. Achour, A. Attar, J. Salfati, P. Pochart, P. Marteau, B. Flourié, F. Bornet and J.-C. Rambaud (1999). "Short-chain fructo-oligosaccharide administration dose-dependently increases fecal bifidobacteria in healthy humans." The Journal of nutrition **129**(1): 113-116.

Buddington, R. K., C. H. Williams, S.-C. Chen and S. Witherly (1996). "Dietary supplement of neosugar alters the fecal flora and decreases activities of some reductive enzymes in human subjects." The American journal of clinical nutrition **63**(5): 709-716.

Buie, T., D. B. Campbell, G. J. Fuchs, 3rd, G. T. Furuta, J. Levy, J. Vandewater, A. H. Whitaker, D. Atkins, M. L. Bauman, A. L. Beaudet, E. G. Carr, M. D. Gershon, S. L. Hyman, P. Jirapinyo, H. Jyonouchi, K. Kooros, R. Kushak, P. Levitt, S. E. Levy, J. D. Lewis, K. F. Murray, M. R. Natowicz, A. Sabra, B. K. Wershil, S. C. Weston, L. Zeltzer and H. Winter (2010). "Evaluation, diagnosis, and treatment of gastrointestinal disorders in individuals with ASDs: a consensus report." Pediatrics **125 Suppl 1**: S1-18.

Chouraqui, J. P., D. Grathwohl, J. M. Labaune, J. M. Hascoet, I. de Montgolfier, M. Leclaire, M. Giarre and P. Steenhout (2008). "Assessment of the safety, tolerance, and protective effect against diarrhea of infant formulas containing mixtures of probiotics or probiotics and prebiotics in a randomized controlled trial." The American journal of clinical nutrition **87**(5): 1365-1373.

Critchfield, J. W., S. van Hemert, M. Ash, L. Mulder and P. Ashwood (2011). "The potential role of probiotics in the management of childhood autism spectrum disorders." Gastroenterol Res Pract **2011**: 161358.
Cunningham-Rundles, S., S. Ahrné, S. Bengmark, R. Johann-Liang, F. Marshall, L. Metakis, C. Califano, A.-M. Dunn, C. Grassey and G. Hinds (2000). "Probiotics and immune response." The American journal of gastroenterology **95**(1): S22-S25.
D'Eufemia, P., M. Celli, R. Finocchiaro, L. Pacifico, L. Viozzi, M. Zaccagnini, E. Cardi and O. Giardini (1996). "Abnormal intestinal permeability in children with autism." Acta Paediatr **85**(9): 1076-1079.
Eiwegger, T., B. Stahl, J. Schmitt, G. Boehm, M. Gerstmayr, J. Pichler, E. Dehlink, C. Loibichler, R. Urbanek and Z. Szépfalusi (2004). "Human milk–derived oligosaccharides and plant-derived oligosaccharides stimulate cytokine production of cord blood T-cells in vitro." Pediatric research **56**(4): 536-540.
Finegold, S. M., S. E. Dowd, V. Gontcharova, C. Liu, K. E. Henley, R. D. Wolcott, E. Youn, P. H. Summanen, D. Granpeesheh, D. Dixon, M. Liu, D. R. Molitoris and J. A. Green, 3rd (2010). "Pyrosequencing study of fecal microflora of autistic and control children." Anaerobe **16**(4): 444-453.
Finegold, S. M., D. Molitoris, Y. Song, C. Liu, M. L. Vaisanen, E. Bolte, M. McTeague, R. Sandler, H. Wexler, E. M. Marlowe, M. D. Collins, P. A. Lawson, P. Summanen, M. Baysallar, T. J. Tomzynski, E. Read, E. Johnson, R. Rolfe, P. Nasir, H. Shah, D. A. Haake, P. Manning and A. Kaul (2002). "Gastrointestinal microflora studies in late-onset autism." Clin Infect Dis **35**(Suppl 1): S6-S16.
Gasser, F. (1994). "Safety of lactic acid bacteria and their occurrence in human clinical infections." Bulletin de l'Institut Pasteur **92**(1): 45-67.
German, J., S. Freeman, C. Lebrilla and D. Mills (2009). "Human milk oligosaccharides: evolution, structures and bioselectivity as substrates for intestinal bacteria."
Gill, H. and J. Prasad (2008). "Probiotics, immunomodulation, and health benefits." Adv Exp Med Biol **606**: 423-454.
Golnik, A. E. and M. Ireland (2009). "Complementary alternative medicine for children with autism: a physician survey." J Autism Dev Disord **39**(7): 996-1005.
Gopal, P. K., J. Prasad and H. S. Gill (2003). "Effects of the consumption of< i> Bifidobacterium lactis</i> HN019 (DR10< sup> TM</sup>) and galacto-oligosaccharides on the microflora of the gastrointestinal tract in human subjects." Nutrition Research **23**(10): 1313-1328.
Horvath, K. and J. A. Perman (2002). "Autism and gastrointestinal symptoms." Curr Gastroenterol Rep **4**(3): 251-258.
Kleessen, B., B. Sykura, H.-J. Zunft and M. Blaut (1997). "Effects of inulin and lactose on fecal microflora, microbial activity, and bowel habit in elderly constipated persons." The American journal of clinical nutrition **65**(5): 1397-1402.
Kruse, H.-P., B. Kleessen and M. Blaut (1999). "Effects of inulin on faecal bifidobacteria in human subjects." British Journal of Nutrition **82**(05): 375-382.
LoCascio, R. G., M. R. Ninonuevo, S. L. Freeman, D. A. Sela, R. Grimm, C. B. Lebrilla, D. A. Mills and J. B. German (2007). "Glycoprofiling of bifidobacterial consumption of human milk oligosaccharides demonstrates strain specific, preferential consumption of small chain glycans secreted in early human lactation." Journal of agricultural and food chemistry **55**(22): 8914-8919.

Mäkeläinen, H., R. Tahvonen, S. Salminen and A. C. Ouwehand (2003). "In vivo safety assessment of two Bifidobacterium longum strains." Microbiology and immunology **47**(12): 911.
Marcobal, A., M. Barboza, J. W. Froehlich, D. E. Block, J. B. German, C. B. Lebrilla and D. A. Mills (2010). "Consumption of human milk oligosaccharides by gut-related microbes." Journal of agricultural and food chemistry **58**(9): 5334-5340.
McFarland, L. V. (2006). "Meta-analysis of probiotics for the prevention of antibiotic associated diarrhea and the treatment of Clostridium difficile disease." The American journal of gastroenterology **101**(4): 812-822.
Merali, Z., S. Lacosta and H. Anisman (1997). "Effects of interleukin-1beta and mild stress on alterations of norepinephrine, dopamine and serotonin neurotransmission: a regional microdialysis study." Brain Res **761**(2): 225-235.
Paineau, D., D. Carcano, G. Leyer, S. Darquy, M. A. Alyanakian, G. Simoneau, J. F. Bergmann, D. Brassart, F. Bornet and A. C. Ouwehand (2008). "Effects of seven potential probiotic strains on specific immune responses in healthy adults: a double-blind, randomized, controlled trial." FEMS Immunol Med Microbiol **53**(1): 107-113.
Parente, F., C. Cucino, A. Anderloni, G. Grandinetti and G. B. Porro (2003). "Treatment of Helicobacter Pylori Infection Using a Novel Antiadhesion Compound (3′ sialyllactose sodium salt). A Double blind, Placebo-Controlled Clinical Study." Helicobacter **8**(4): 252-256.
Pedersen, A., B. Sandström and J. M. Van Amelsvoort (1997). "The effect of ingestion of inulin on blood lipids and gastrointestinal symptoms in healthy females." British Journal of Nutrition **78**(02): 215-222.
Puccio, G., C. Cajozzo, F. Meli, F. Rochat, D. Grathwohl and P. Steenhout (2007). "Clinical evaluation of a new starter formula for infants containing live< i> Bifidobacterium longum</i> BL999 and prebiotics." Nutrition **23**(1): 1-8.
Saxelin, M., N. H. Chuang, B. Chassy, H. Rautelin, P. H. Makela, S. Salminen and S. L. Gorbach (1996). "Lactobacilli and bacteremia in southern Finland, 1989-1992." Clin Infect Dis **22**(3): 564-566.
Sela, D., J. Chapman, A. Adeuya, J. Kim, F. Chen, T. Whitehead, A. Lapidus, D. Rokhsar, C. Lebrilla and J. German (2008). "The genome sequence of Bifidobacterium longum subsp. infantis reveals adaptations for milk utilization within the infant microbiome." Proceedings of the National Academy of Sciences **105**(48): 18964-18969.
Sela, D. A., Y. Li, L. Lerno, S. Wu, A. M. Marcobal, J. B. German, X. Chen, C. B. Lebrilla and D. A. Mills (2011). "An infant-associated bacterial commensal utilizes breast milk sialyloligosaccharides." Journal of Biological Chemistry **286**(14): 11909-11918.
Silk, D., A. Davis, J. Vulevic, G. Tzortzis and G. Gibson (2008). "Clinical trial: the effects of a trans-galactooligosaccharide prebiotic on faecal microbiota and symptoms in irritable bowel syndrome." Alimentary pharmacology & therapeutics **29**(5): 508-518.
Wolf, B., K. Wheeler, D. Ataya and K. Garleb (1998). "Safety and tolerance of< i> Lactobacillus reuteri</i> supplementation to a population infected with the human immunodeficiency virus." Food and chemical toxicology **36**(12): 1085-1094.
Ziegler, E., J. A. Vanderhoof, B. Petschow, S. H. Mitmesser, S. I. Stolz, C. L. Harris and C. L. Berseth (2007). "Term infants fed formula supplemented with selected blends of prebiotics grow normally

and have soft stools similar to those reported for breast-fed infants." Journal of pediatric gastroenterology and nutrition **44**(3): 359-364.
Zivkovic, A. M., J. B. German, C. B. Lebrilla and D. A. Mills (2011). "Human milk glycobiome and its impact on the infant gastrointestinal microbiota." Proceedings of the National Academy of Sciences **108**(Supplement 1): 4653-4658.
Zoppi, G., M. Cinquetti, A. Benini, E. Bonamini and E. B. Minelli (2001). "Modulation of the intestinal ecosystem by probiotics and lactulose in children during treatment with ceftriaxone." Current therapeutic research **62**(5): 418-435.
